# A High-Throughput Size Exclusion Chromatography Method to Determine the Molecular Size Distribution of Meningococcal Polysaccharide Vaccine

**DOI:** 10.1155/2016/9404068

**Published:** 2016-09-05

**Authors:** Imran Khan, K. M. Taufiqur Rahman, S. M. Saad Us Siraj, Mahbubul Karim, Abdul Muktadir, Arpan Maheshwari, Md Azizul Kabir, Zebun Nahar, Mohammad Mainul Ahasan

**Affiliations:** Incepta Vaccine Ltd., Zirabo, Savar, Dhaka 1341, Bangladesh

## Abstract

Molecular size distribution of meningococcal polysaccharide vaccine is a readily identifiable parameter that directly correlates with the immunogenicity. In this paper, we report a size exclusion chromatography method to determine the molecular size distribution and distribution coefficient value of meningococcal polysaccharide serogroups A, C, W, and Y in meningococcal polysaccharide (ACWY) vaccines. The analyses were performed on a XK16/70 column packed with sepharose CL-4B with six different batches of Ingovax® ACWY, a meningococcal polysaccharide vaccine produced by Incepta Vaccine Ltd., Bangladesh. A quantitative rocket immunoelectrophoresis assay was employed to determine the polysaccharide contents of each serogroup. The calculated distribution coefficient values of serogroups A, C, W, and Y were found to be 0.26 ± 0.16, 0.21 ± 0.11, 0.21 ± 0.11, and 0.14 ± 0.12, respectively, and met the requirements of British Pharmacopeia. The method was proved to be robust for determining the distribution coefficient values which is an obligatory requirement for vaccine lot release.

## 1. Introduction 

*Neisseria meningitidis* is the prime cause of bacterial meningitis and fatal sepsis in human [1, 2]. It has been a serious public health threat worldwide due to higher rates of morbidity and mortality [3, 4]. Only 6 (A, B, C, W, X, and Y) of 13 immunologically distinct serogroups are clinically relevant and responsible for invasive meningococcal disease [5]. In most cases, serogroups A, C, W, and Y are the main causes of epidemic meningitis and protective vaccines are available against these serogroups [6]. The relative dispersion of each serogroup varies over time, ethnicity, geographical location, and, in some cases, socioeconomic factors [7–9]. In African “meningitis belt” the magnitude outbreak was surveilled for serogroups A and C [10]. Serogroup W was a menace for Hajj pilgrims in 2000 and 2001 [11–13]. In recent time, the epidemiological surveillance showed that serogroup Y meningococcal disease has rapidly increased in some parts of Europe [14]. India, Indonesia, Mongolia, Nepal, Pakistan, and Vietnam have experienced a repeated occurrence of serogroup A (or C in Vietnam) meningococcal epidemics in last 30 years [15]. In Bangladesh, bacterial meningitis from meningococcal infection causes a significant morbidity and mortality in adults and children [16, 17]. It was reported in a study of bacterial meningitis that 136 of 189 diagnosed patients had* Neisseria meningitidis* group A or C infection in Bangladesh. It was also claimed that the case-fatality ratio for these meningococcal infections was 10%.

Capsular polysaccharides of* N. meningitidis* are immunogenic and used as vaccine against meningococcal diseases. The meningococcal polysaccharide ACWY (PS) vaccine is derived from capsular polysaccharides of meningococci group A (*O*-acetylated repeating units of* N*-acetylmannosamine, linked with 1*α* → 6 phosphodiester bonds), C (*O*-acetylated repeating units of sialic acid, linked with 2*α* → 9 glycosidic bonds), W (*O*-acetylated alternating units of sialic acid and D-galactose, linked with 2*α* → 6 and 1*α* → 4 glycosidic bonds), and Y (*O*-acetylated alternating units of sialic acid and D-glucose, linked with 2*α* → 6 and 1*α* → 4 glycosidic bonds) [18, 19]. In particular, the efficacy of a PS vaccine is dependent on specific immunochemical and physical properties. The molecular size of polysaccharide is one of the major parameters which can be directly correlated with vaccine immunogenicity and potency [20–22]. This parameter is directly involved in inducing long-lasting immune memory [23]. Moreover, during manufacturing steps it is crucial for routinely monitored batch-to-batch consistency [20–22].

The idea of molecular size based separation by chromatography, also known as size exclusion chromatography (SEC), was first introduced by Synge [24]. It is a widely used technique for determining the molecular size distribution (MSD) of polymeric materials (e.g., proteins, nucleic acids, and polysaccharides) [25]. Chromatography media selection is indispensable for determination of molecular size distribution (MSD) due to the variability in media specifications [20]. The MSD of bacterial capsular polysaccharide is determined by using either only sepharose or cross-linked sepharose (e.g., sepharose CL-4B) [26]. McCauley and his colleagues suggested that sepharose CL-2B performed better than sepharose for MSD [27]. Studies with sepharose CL-4B (a 4% cross-linked agarose matrix) were reported to be better than both sepharose CL-2B [28] and sepharose 6B in regard to performance [29].

Meningococcal polysaccharide can be estimated by assaying either phosphorus content (for serogroup A) or sialic acid content (for serogroups C, Y, and W) [19, 30]. However, it is difficult to estimate serospecific polysaccharide content by above-mentioned assays from a mixture of ACWY polysaccharides in tetravalent meningococcal PS vaccine. Immunochemical assays could be suitable platform to estimate individual serospecific polysaccharide from a mixture of polysaccharide. It has been appraised that a reliable and quantitative immunochemical method would be crucial for the estimation of the PS content. In this connection, British Pharmacopeia (BP) [19] and World Health Organization (WHO) [26] have listed the rocket immunoelectrophoresis as a widely used tool.

Analysis of MSD of individual polysaccharide in multivalent vaccine is always challenging. A rapid and reproducible method for estimation of MSD of meningococcal tetravalent vaccine of ACWY may help vaccine researcher from academy as well as industry to develop effective multivalent vaccines. In the present investigation, we described a SEC method for determination of MSD of tetravalent meningococcal polysaccharide vaccine of ACWY. This experiment mainly focused on calculation and comparative analysis of distribution coefficient (*K*_*D*_) value of each serogroup of six different batches of meningococcal PS vaccines.

## 2. Experimental

### 2.1. Meningococcal PS Vaccine Preparation

Ingovax ACWY, a tetravalent meningococcal polysaccharide vaccine produced by Incepta Vaccine Ltd., Bangladesh, was used in this study. It is a freeze-dried vaccine containing ≥50 *μ*g of each of the polysaccharide antigens from serogroups A, C, W, and Y. Six batches of Ingovax ACWY vaccines were tested to determine the elution profile of each polysaccharide serogroup. From each batch, 40 vials containing ≥2.0 mg of PS were reconstituted in 2.5 mL of 0.2 M NaCl (pH 7.39, cond. 21.2 mS/cm) for analysis.

### 2.2. Chromatography Column Preparation

XK16/70 (GE Healthcare) column (porosity 10 *μ*m) was used in this study. The column packed with sepharose CL-4B (particle size 45 *μ*m–165 *μ*m) (GE Healthcare). These cross-linked bead formed agarose matrix particles have an exclusion limit between 70 and 20,000 KDa. All the chromatography operation was conducted at temperature of 23 ± 2°C.

### 2.3. Size Exclusion Chromatography (SEC)

**Figure 1:**
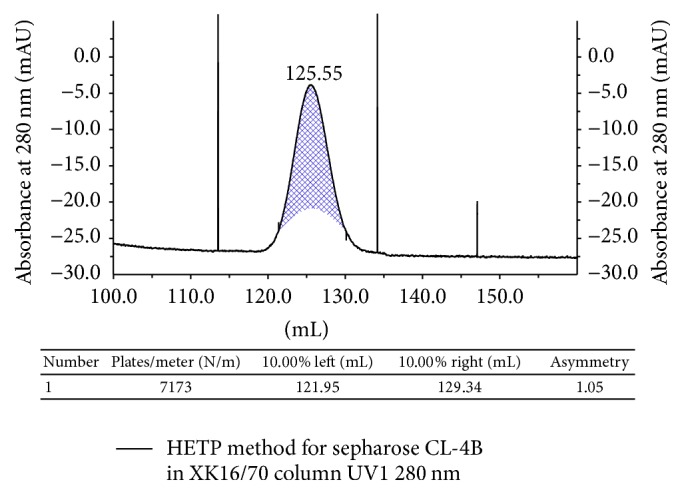
Performance testing of XK16/70 packed with sepharose CL-4B column.

SEC was performed for MSD analysis by AKTA purifier 100 (GE Healthcare). The Unicorn 5.2 software package was used for programming method run and data analysis. The column performance test was conducted by injecting 200 *μ*L of 2% (v/v) acetone (Sigma) through the column using purified water as eluent. By Unicorn software the packed column theoretical plate number was calculated as 7173 plates/m and peak asymmetry factor was found 1.05 which matches the acceptance criteria of sepharose CL-4B for column use (Figure 1).

The column was equilibrated with 1.5 column volume (CV) equal to 187.5 mL (1 CV = 125 mL) of 0.2 M NaCl (pH 7.39, cond. 21.2 mS/cm, temp. 25°C). 2 mL of reconstituted vaccine samples (using 2 mL sample loop) was injected and run with a flow rate of 0.3 mL/min. The automatic fraction collector was applied to collect fixed volume of 2 mL fractions. The fractions were stored at −20°C until further test.

### 2.4. Determination of *K*_*D*_

The *K*_*D*_ values for Ingovax ACWY vaccines were calculated according to Ackers' formula [31, 32],

(1) $$\begin{array}{c} K_{D}=\frac{\left(V_{e}-V_{o}\right)}{\left(V_{t}-V_{o}\right)}, \end{array}$$

where *V*_*o*_ is void volume, *V*_*t*_ is total bed volume, and *V*_*e*_ is elution volume.

**Figure 2:**
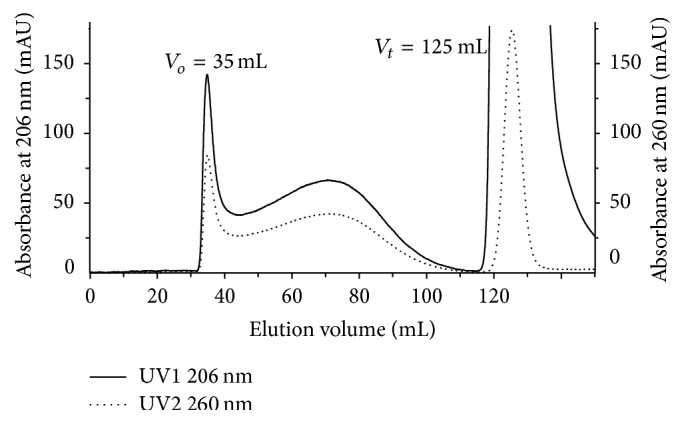
The elution profile of blue dextran 2000 and sodium azide. The absorbance of blue dextran 2000 was monitored at 206 nm and sodium azide at 260 nm.

Here, *V*_*o*_ interprets the exterior part of the beads and is determined by using a molecule which is larger than the exclusion limit of a particular matrix. Small molecule elutes at the end of the column and is determined as *V*_*t*_. In this method, *V*_*o*_ and *V*_*t*_ were determined by 0.2% (w/v) blue dextran 2000, a polysaccharide covalently bonded with blue dye, MW 2000 KDa (GE Healthcare), and 0.5% (w/v) sodium azide (MW 65 Da) (Sigma), respectively. The absorbances for blue dextran 2000 and sodium azide were monitored at 206 nm and 260 nm, respectively (Figure 2). The elution volume (*V*_*e*_) was calculated by estimating the principal peak of polysaccharides. In this experiment, we have also calculated the recovered percentage of eluted polysaccharide before *K*_*D*_ value 0.50 by following formula:

(2) %  of  recovered  polysaccharide  before  KD  0.5=Recovered  polysaccharide  of  the  group  upto  elution  volume  80 mLLoaded  polysaccharide  of  the  group×100.

### 2.5. Rocket Immunoelectrophoresis

Rocket immunoelectrophoresis (RIE) was used for determination of polysaccharide content in each serogroup. Briefly, agarose (1%) (HiMedia) was melted in 20 mL of 0.1 M Tris buffer, pH 8.2 by boiling in heating mantle. Liquid agarose was cooled to 50–55°C and 200 *μ*L of* Neisseria meningitidis* Antiserum (BD) was added and mixed gently. The gel was immediately poured onto a gel tray placed on a horizontal surface. The gel was allowed to solidify and 3 mm sample wells were made using gel-puncher. The gel was set in the electrophoresis unit. The reference standard (NIBSC, UK) solution was 100 *μ*g/mL of polysaccharide in 0.1 M Tris buffer, pH 8.2. The total amounts of samples were distributed in 8 different pools for our experiment suitability. 8 *μ*L of samples and standards were directly loaded to the well in duplicate. Electrophoretic run was conducted for 15 hr at 9 mA. The gel was stained by staining solution containing Coomassie brilliant blue 0.1% (w/v), methanol 45% (v/v), and glacial acetic acid 10% (v/v) for 40 minutes using Mini-Orbital Shaker with gentle agitation, and destained in destaining solution containing acetic acid 7% (v/v) and methanol 10% (v/v).

The polysaccharide content was calculated by following formula:

(3) Polysaccharide  content=Sample  height×Average  standard  concentrationAverage  standard  height.

## 3. Results

**Figure 3:**
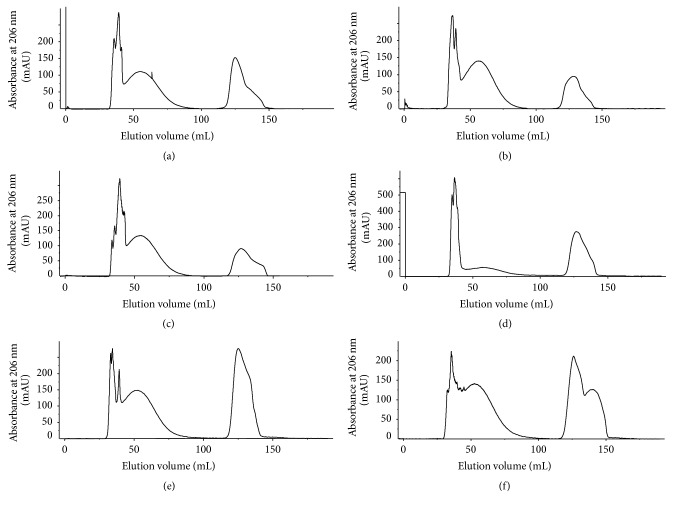
Elution profile of six batches of Ingovax ACWY vaccine. Chromatograms of batches (a) 15001, (b) 15002, (c) 15003, (d) 15004, (e) 15005, and (f) 15006 are shown. The peak absorbance was monitored at 206 nm.

In this study, we determined *K*_*D*_ value of meningococcal polysaccharide serogroups A, C, W, and Y in meningococcal PS vaccine. For this purpose, *V*_*o*_ and *V*_*t*_ were determined by blue dextran 2000 and sodium azide, respectively. Figure 1 shows their corresponding values as 35 mL and 125 mL, respectively. The elution profile of individual six batches of Ingovax ACWY was considered for MSD analysis. Serogroups A, C, W, and Y showed symmetric peak at absorbance 206 nm (Figure 3). The fraction volumes were collected row by row in a 2 mL volume using automated fraction collector and polysaccharides content of individual serogroup was determined by rocket immunoelectrophoresis (see S1 and S2 in Supplementary Material available online at http://dx.doi.org/10.1155/2016/9404068). The chromatogram indicated that the peak started from 35 mL in all six batches (Figures 3(a)–3(f)).

Result of RIE of eluted fractions showed that the principal peak fractions appeared for serogroups A, C, W, and Y at fractions C10, C5, C5, and B7, respectively, in the batch 15001; D1, C7, C7, and C7, respectively, in the batch 15002; C10, C5, C5, and C5, respectively, in the batch 15003; and C5, C5, C5, and B10, respectively in the batch 15005. However, in case of batches 15004 and 15006 the principal peak fractions appeared for all serogroups at fractions B7 and B11, respectively.

**Figure 4:**
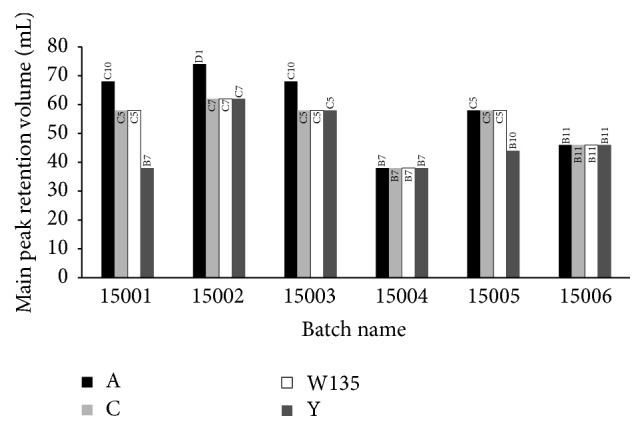
Principal peak fraction volume of six batches of Ingovax ACWY vaccine.

At batch 15001, the principle peak of meningococcal serogroup A was observed at fraction C10 with *V*_*e*_ of 68 mL. For serogroup C, the principal peak was found at C5 (*V*_*e*_ = 58 mL). In case of serogroups W and Y, the principal peak was at C5 (*V*_*e*_ = 58 mL) and B7 (*V*_*e*_ = 38 mL), respectively. The *K*_*D*_ values for serogroups A, C, W, and Y were calculated using Ackers' formula [31, 32] and the values were found to be 0.37, 0.26, 0.26, and 0.03, respectively (Figure 4; Table 1). However, the principal peak for serogroup A was observed at fraction D1 (*V*_*e*_ = 74 mL) at batch 15002. For serogroups C, W, and Y, the principal peak was found at the same fraction C7 (*V*_*e*_ = 62 mL). The *K*_*D*_ value for serogroups C, W, and Y was 0.30 while it was 0.43 for serogroup A at this batch. The elution patterns of batches 15001, 15002, 15003, and 15005 were indistinguishable from each other. However, it was slightly different at batches 15004 and 15006. Interestingly, all the principal peaks appeared at the same position for each serogroup at batches 15004 (B7, *V*_*e*_ = 38 mL) and 15006 (B11, *V*_*e*_ = 46 mL), though the elution patterns were found to be different. The *K*_*D*_ values were 0.03 for batch 15004 and 0.12 for batch 15006. In the cases of batches 15001, 15003, and 15005, the principal peak appeared at fraction C5 (*V*_*e*_ = 58 mL) for both serogroups C and W while the *K*_*D*_ value was found to be 0.26. Besides, for serogroup A the principal peak was seen at fraction C10 (*V*_*e*_ = 68 mL) with *K*_*D*_ value of 0.37 at batches 15001 and 15003. The mean *K*_*D*_ ± SD of serogroups A, C, W, and Y, derived from the analysis of six different batches, corresponded to 0.26 ± 0.16, 0.21 ± 0.11, 0.21 ± 0.11, and 0.14 ± 0.12, respectively.

Additionally, we calculated the total recovery percentage of polysaccharides eluted before *K*_*D*_ value 0.5 for each serogroup of six batches. The mean ± SD recovery percentages of A, C, W, and Y were 90.78 ± 8.11, 91.50 ± 5.89, 86.64 ± 6.42, and 84.05 ± 2.74, respectively. The lowest recovery of serogroups A, C, and W was found in batch 15004 (80.75%, 84.42%, and 80.53%, resp.) and in case of Y it was found in batch 15001 (81.16%) (Table 2). Each of the individual serospecific polysaccharides for each batch complies with the requirements of BP [19].

## 4. Discussions

The MSD is a key indicator for process consistency, confirming the vaccines immunogenicity and safety [33–37]. Therefore, a robust and reliable method for determination of MSD of meningococcal PS vaccine is crucial. von Hunolstein and her coauthors [21] reported the experimental conditions for SEC on a tandem equipped PL Aquagel-OH 60 column (7.5 × 300 mm) and PL Aquagel-OH guard column (50 mm × 7.5 mm) for MSD analysis of meningococcal PS vaccine. In this study, we described a robust SEC method for MSD analysis of meningococcal PS vaccine on a XK16/70 column packed with sepharose CL-4B.

We identified *K*_*D*_ values of six batches of Ingovax ACWY vaccine and analysed the batch-to-batch fluctuations. The lower *K*_*D*_ value indicates the higher molecular size of polysaccharide which suggests the polysaccharide to be more immunogenic [25, 38]. In the present investigation, very little variance was observed amongst the batches. This indicates that the method was accurate and reproducible. Again, *K*_*D*_ values of individual serogroups of six batches were compared where lowest values were found in all serogroups at batches 15004 and 15006 while the highest values were found at batch 15002. This can be explained from the fact that determination of absolute molecular size distribution is difficult as a result of polydispersity ranging from 5 × 10^4^ to 2 × 10^5^ Da [26]. Therefore, some fluctuations in *K*_*D*_ values could be considered insignificant. Misplay of formulation and filling may affect the structural integrity and size of polysaccharides [30] and hydrolysis reduces the molecular size of polysaccharide which may deteriorate the immunogenic capability [26, 39]. In our study, the polysaccharides were stored in −20°C to reduce the possibility of such degradation.

According to the requirements of BP [19], the *K*_*D*_ value for the principal peak should not be greater than 0.70 for serogroups A and C; 0.57 for serogroup Y; and 0.68 for serogroup W for meningococcal PS vaccine. In our study, the highest values of serogroups A, C, W, and Y were found to be 0.43, 0.30, 0.30, and 0.30, respectively in batch 15002. Therefore, the *K*_*D*_ values of each serogroup of six batches of Ingovax ACWY vaccine comply with the requirements of BP [19]. Additionally, our method can be applied to calculate recovery percentage of polysaccharides of individual meningococcal serogroups. According to requirement of BP [19], at least 65% polysaccharide of serogroup A, 75% of serogroup C, 80% of serogroup W, and 80% of serogroup Y should be eluted before *K*_*D*_ value 0.50. In this study, it is found that all the study batches of Ingovax ACWY also meet the requirements of BP [19] for recovery of individual polysaccharide (Table 2). Overall results of this experiment showed that Ingovax ACWY was highly immunogenic and could serve as a potential meningococcal PS vaccine for human use in compliance with BP [19] and WHO [26].

## 5. Conclusion

SEC could be employed successfully for MSD analysis of polysaccharide vaccine and meaningful information in regard to reproducibility and accuracy of a method could be extracted towards fulfilling the requirements of regulatory authority's approval of a vaccine for human use. The present study reports for the first time the use of SEC on a XK16/70 column packed with sepharose CL-4B for the determination of *K*_*D*_ values of different serogroups of meningococcal polysaccharide vaccine, as an effective means of indicating reproducibility, accuracy, and performance at industrial settings.

## Supplementary Material

S1: The polysaccharide content of all fractions was calculated from the value of rocket height of samples & standards and standard's concentrations. S2: The total percentage of recovered polysaccharide for all serogroups was shown.

## Figures and Tables

**Table 1:** Main peak fraction volumes and corresponding *K*_*D*_ values of six batches of Ingovax ACWY vaccine (the highest *K*_*D*_ values were indicated as bold and lowest ones as bold italic letters).

Batch number	Serogroup A		Serogroup C		Serogroup W		Serogroup Y	
	Main peak fraction, mL	*K*_*D*_	Main peak fraction, mL	*K*_*D*_	Main peak fraction, mL	*K*_*D*_	Main peak fraction, mL	*K*_*D*_
15001	68	0.37	58	0.26	58	0.26	38	0.03
15002	74	**0.43**	62	**0.30**	62	**0.30**	62	**0.30**
15003	68	0.37	58	0.26	58	0.26	58	0.26
15004	38	***0.03***	38	***0.03***	38	***0.03***	38	***0.03***
15005	58	0.26	58	0.26	58	0.26	44	0.10
15006	46	***0.12***	46	***0.12***	46	***0.12***	46	***0.12***

**Table 2:** The injected and recovered polysaccharide content of serogroups A, C, Y, and W of six batches of Ingovax ACWY vaccine.

Batch number	Serogroup A		Serogroup C		Serogroup W		Serogroup Y	
	Injected (*μ*g)	Recovered (% before *K*_*D*_ 0.5)	Injected (*μ*g)	Recovered (% before *K*_*D*_ 0.5)	Injected (*μ*g)	Recovered (% before *K*_*D*_ 0.5)	Injected (*μ*g)	Recovered (% before *K*_*D*_ 0.5)
15001	1921.73	98.37	1918.5	97.31	2097.17	81.15	2121.49	81.16
15002	1919.49	98.56	2067.17	97.07	2054.43	96.23	2229.44	84.81
15003	1975.04	97.12	2163.87	88.85	2000.19	92.22	2084.93	86.73
15004	2098.08	80.75	1924.0	84.42	2075.58	80.53	2122.78	82.10
15005	2028.51	84.00	1908.48	95.67	2059.1	82.75	2146.75	81.88
15006	2059.74	85.86	1941.50	85.66	2070.5	86.96	2092.32	87.64
