# Supplementary material for: A High-Throughput Size Exclusion Chromatography Method to Determine the Molecular Size Distribution of Meningococcal Polysaccharide Vaccine
Source: Int J Anal Chem. 2016 Sep 5;2016:9404068. doi: 10.1155/2016/9404068 (PMC5027369; doi:10.1155/2016/9404068)
Supplement: Supplementary file 1 — S1: The polysaccharide content of all fractions was calculated from the value of rocket height of samples & standards and standard's concentrations. S2: The total percentage of recovered polysaccharide for all serogroups was shown. [file 9404068.f1.zip › S1.docx]

**S1: Determination of meningococcal polysaccharide (PS) contents of six batches of Ingovax®ACWY by Rocket Immunoelectrophoresis.**

**Batch: 15001**

**Table: Determination of Meningococcal Polysaccharide content (Group-A )**

| **Well No.** | **Name of Standard** | **Rocket Height (mm )** | ***Concentration (µg)** |
| --- | --- | --- | --- |
| 1 | Std-0.5 | 11.09 | 0.05 |
| 2 | Std-1 | 13.84 | 0.1 |
| 3 | Std-2 | 16.73 | 0.2 |
| 4 | Std-4 | 18.96 | 0.4 |
| 5 | Std-8 | 21.54 | 0.8 |
|  | | | |
| **Sl. NO.** | **Sample Pool No.** | **Rocket Height (mm )** | **Total Polysaccharide, µg** |
| 1 | Pool-1 | 0 | 0 |
| 2 | Pool-2 | 11.21 | 264.25 |
| 3 | Pool-3 | 13.97 | 328.75 |
| 4 | Pool-4 | 16.80 | 396.125 |
| 5 | Pool-5 | 18.63 | 439.25 |
| 6 | Pool-6 | 9.34 | 264 |
| 7 | Pool-7 | 4.71 | 198 |
| 8 | Pool-8 | 0 | 0 |

Average standard concentration = 0.31 µg

Average standard height = 16.432 mm

**Calculation:**

*Polysaccharide content/8 µl= (Sample height × Average standard concentration)/Average standard height

(Polysaccharide content / 8 µl ) × Total volume of sample pool

*Polysaccharide content /sample pool =

8

**Table: Determination of Meningococcal Polysaccharide content (Group-C )**

| **Well No.** | **Name of Standard** | **Rocket Height (mm )** | ***Concentration (µg)** |
| --- | --- | --- | --- |
| 1 | Std-1 | 10.34 | 0.1 |
| 2 | Std-2 | 16.05 | 0.2 |
| 3 | Std-4 | 19.02 | 0.4 |
| 4 | Std-8 | 22.60 | 0.8 |
|  | | | |
| **Sl. NO.** | **Sample Pool No.** | **Rocket Height (mm )** | **Total Polysaccharide, µg** |
| 1 | Pool-1 | 0 | 0 |
| 2 | Pool-2 | 13.82 | 381 |
| 3 | Pool-3 | 13.87 | 382.375 |
| 4 | Pool-4 | 17.78 | 490.125 |
| 5 | Pool-5 | 13.81 | 380.625 |
| 6 | Pool-6 | 7.04 | 232.8 |
| 7 | Pool-7 | 0 | 0 |
| 8 | Pool-8 | 0 | 0 |

Average standard concentration = 0.375 µg

Average standard height = 17.0025 mm

**Table: Determination of Meningococcal Polysaccharide content (Group-W)**

| **Well No.** | **Name of Standard** | **Rocket Height (mm )** | ***Concentration (µg)** |
| --- | --- | --- | --- |
| 1 | Std-1 | 17.34 | 0.1 |
| 2 | Std-2 | 19.09 | 0.2 |
| 3 | Std-4 | 20.89 | 0.4 |
| 4 | Std-8 | 22.46 | 0.8 |
|  | | | |
| **Sl. NO.** | **Sample Pool No.** | **Rocket Height (mm )** | **Total Polysaccharide, µg** |
| 1 | Pool-1 | 0 | 0 |
| 2 | Pool-2 | 16.53 | 388.375 |
| 3 | Pool-3 | 15.86 | 372.625 |
| 4 | Pool-4 | 16.63 | 390.75 |
| 5 | Pool-5 | 15.72 | 369.375 |
| 6 | Pool-6 | 6.41 | 180.75 |
| 7 | Pool-7 | 0.85 | 35.775 |
| 8 | Pool-8 | 0 | 0 |

Average standard concentration = 0.375 µg

Average standard height = 19.945 mm

**Table: Determination of Meningococcal Polysaccharide content (Group-Y)**

| **Well No.** | **Name of Standard** | **Rocket Height (mm )** | ***Concentration (µg)** |
| --- | --- | --- | --- |
| 1 | Std-0.5 | 6.72 | 0.05 |
| 2 | Std-1 | 9.01 | 0.1 |
| 3 | Std-2 | 11.31 | 0.2 |
| 4 | Std-4 | 13.63 | 0.4 |
| 5 | Std-8 | 15.26 | 0.8 |
|  | | | |
| **Sl. NO.** | **Sample Pool No.** | **Rocket Height (mm )** | **Total Polysaccharide, µg** |
| 1 | Pool-1 | 0 | 0 |
| 2 | Pool-2 | 12.67 | 438.75 |
| 3 | Pool-3 | 11.74 | 406.025 |
| 4 | Pool-4 | 11.30 | 391.375 |
| 5 | Pool-5 | 9.81 | 339.75 |
| 6 | Pool-6 | 3.5 | 145.35 |
| 7 | Pool-7 | 0.54 | 33.525 |
| 8 | Pool-8 | 0 | 0 |
| 9 | Pool-9 | 0 | 0 |

Average standard concentration = 0.31 µg

Average standard height = 11.186 mm

**Batch: 15002**

**Table: Determination of Meningococcal Polysaccharide content (Group-A )**

| **Well No.** | **Name of Standard** | **Rocket Height (mm )** | ***Concentration (µg)** |
| --- | --- | --- | --- |
| 1 | Std-0.5 | 13.93 | 0.05 |
| 2 | Std-1 | 16.69 | 0.1 |
| 3 | Std-2 | 19.04 | 0.2 |
| 4 | Std-4 | 22.17 | 0.4 |
| 5 | Std-8 | 24.98 | 0.8 |
|  | | | |
| **Sl. NO.** | **Sample Pool No.** | **Rocket Height (mm )** | **Total Polysaccharide, µg** |
| 1 | Pool-1 | 0 | 0 |
| 2 | Pool-2 | 14.09 | 281.25 |
| 3 | Pool-3 | 14.83 | 415.45 |
| 4 | Pool-4 | 17.03 | 409.5 |
| 5 | Pool-5 | 18.82 | 451.95 |
| 6 | Pool-6 | 9.56 | 191.25 |
| 7 | Pool-7 | 5.09 | 142.45 |
| 8 | Pool-8 | 0 | 0 |
| 9 | Pool-9 | 0 | 0 |

Average standard concentration = 0.31 µg

Average standard height = 19.362 mm

**Table: Determination of Meningococcal Polysaccharide content (Group-C )**

| **Well No.** | **Name of Standard** | **Rocket Height (mm )** | ***Concentration (µg)** |
| --- | --- | --- | --- |
| 1 | Std-1 | 10.13 | 0.1 |
| 2 | Std-2 | 12.32 | 0.2 |
| 3 | Std-4 | 13.87 | 0.4 |
| 4 | Std-8 | 15.39 | 0.8 |
|  | | | |
| **Sl. NO.** | **Sample Pool No.** | **Rocket Height (mm )** | **Total Polysaccharide, µg** |
| 1 | Pool-1 | 0 | 0 |
| 2 | Pool-2 | 8.13 | 293.75 |
| 3 | Pool-3 | 10.79 | 546 |
| 4 | Pool-4 | 13.08 | 568.5 |
| 5 | Pool-5 | 10.03 | 435 |
| 6 | Pool-6 | 3.12 | 112.5 |
| 7 | Pool-7 | 1.01 | 50.75 |
| 8 | Pool-8 | 0 | 0 |

Average standard concentration = 0.375 µg

Average standard height = 12.9275 mm

**Table: Determination of Meningococcal Polysaccharide content (Group-W135)**

| **Well No.** | **Name of Standard** | **Rocket Height (mm )** | ***Concentration (µg)** |
| --- | --- | --- | --- |
| 1 | Std-1 | 16.87 | 0.1 |
| 2 | Std-2 | 18.28 | 0.2 |
| 3 | Std-4 | 19.98 | 0.4 |
| 4 | Std-8 | 20.96 | 0.8 |
|  | | | |
| **Sl. NO.** | **Sample Pool No.** | **Rocket Height (mm )** | **Total Polysaccharide, µg** |
| 1 | Pool-1 | 0 | 0 |
| 2 | Pool-2 | 14.93 | 367.5 |
| 3 | Pool-3 | 15.84 | 546 |
| 4 | Pool-4 | 18.01 | 532 |
| 5 | Pool-5 | 13.75 | 406.59 |
| 6 | Pool-6 | 3.78 | 93.125 |
| 7 | Pool-7 | 0.91 | 31.325 |
| 8 | Pool-8 | 0 | 0 |

Average standard concentration = 0.375 µg

Average standard height = 19.0225 mm

**Table: Determination of Meningococcal Polysaccharide content (Group-Y)**

| **Well No.** | **Name of Standard** | **Rocket Height (mm )** | ***Concentration (µg)** |
| --- | --- | --- | --- |
| 1 | Std-0.5 | 6.98 | 0.05 |
| 2 | Std-1 | 9.99 | 0.1 |
| 3 | Std-2 | 11.87 | 0.2 |
| 4 | Std-4 | 14.61 | 0.4 |
| 5 | Std-8 | 17.03 | 0.8 |
|  | | | |
| **Sl. NO.** | **Sample Pool No.** | **Rocket Height (mm )** | **Total Polysaccharide, µg** |
| 1 | Pool-1 | 0 | 0 |
| 2 | Pool-2 | 6.63 | 211.25 |
| 3 | Pool-3 | 12.01 | 537.25 |
| 4 | Pool-4 | 14.97 | 574.5 |
| 5 | Pool-5 | 10.15 | 390 |
| 6 | Pool-6 | 4.33 | 138.71 |
| 7 | Pool-7 | 0.93 | 41.65 |
| 8 | Pool-8 | 0 | 0 |
| 9 | Pool-9 | 0 | 0 |

Average standard concentration = 0.31 µg

Average standard height = 12.096 mm

**Batch: 15003**

**Table: Determination of Meningococcal Polysaccharide content (Group-A )**

| **Well No.** | **Name of Standard** | **Rocket Height (mm )** | ***Concentration (µg)** |
| --- | --- | --- | --- |
| 1 | Std-0.5 | 13.065 | 0.05 |
| 2 | Std-1 | 15.325 | 0.1 |
| 3 | Std-2 | 17.896 | 0.2 |
| 4 | Std-4 | 21.012 | 0.4 |
| 5 | Std-8 | 23.731 | 0.8 |
|  | | | |
| **Sl. NO.** | **Sample Pool No.** | **Rocket Height (mm )** | **Total Polysaccharide, µg** |
| 1 | Pool-1 | 0 | 0 |
| 2 | Pool-2 | 13.68 | 290 |
| 3 | Pool-3 | 14.01 | 297.5 |
| 4 | Pool-4 | 15.87 | 337.5 |
| 5 | Pool-5 | 17.92 | 381.25 |
| 6 | Pool-6 | 15.51 | 396 |
| 7 | Pool-7 | 5.64 | 216 |
| 8 | Pool-8 | 0 | 0 |
| 9 | Pool-9 | 0 | 0 |

Average standard concentration = 0.31 µg

Average standard height = 18.205 mm

**Table: Determination of Meningococcal Polysaccharide content (Group-C )**

| **Well No.** | **Name of Standard** | **Rocket Height (mm )** | ***Concentration (µg)** |
| --- | --- | --- | --- |
| 1 | Std-1 | 10.27 | 0.1 |
| 2 | Std-2 | 13.38 | 0.2 |
| 3 | Std-4 | 16.61 | 0.4 |
| 4 | Std-8 | 20.04 | 0.8 |
|  | | | |
| **Sl. NO.** | **Sample Pool No.** | **Rocket Height (mm )** | **Total Polysaccharide, µg** |
| 1 | Pool-1 | 0 | 0 |
| 2 | Pool-2 | 12.13 | 376.25 |
| 3 | Pool-3 | 13.69 | 425 |
| 4 | Pool-4 | 15.13 | 470 |
| 5 | Pool-5 | 12.87 | 400 |
| 6 | Pool-6 | 5.13 | 190.5 |
| 7 | Pool-7 | 1.09 | 60.75 |
| 8 | Pool-8 | 0 | 0 |

Average standard concentration = 0.375 µg

Average standard height = 15.075 mm

**Table: Determination of Meningococcal Polysaccharide content (Group-W135)**

| **Well No.** | **Name of Standard** | **Rocket Height (mm )** | ***Concentration (µg)** |
| --- | --- | --- | --- |
| 1 | Std-1 | 17.24 | 0.1 |
| 2 | Std-2 | 18.60 | 0.2 |
| 3 | Std-4 | 20.02 | 0.4 |
| 4 | Std-8 | 21.87 | 0.8 |
|  | | | |
| **Sl. NO.** | **Sample Pool No.** | **Rocket Height (mm )** | **Total Polysaccharide, µg** |
| 1 | Pool-1 | 0 | 0 |
| 2 | Pool-2 | 15.82 | 381.25 |
| 3 | Pool-3 | 17.97 | 432.5 |
| 4 | Pool-4 | 18.04 | 435 |
| 5 | Pool-5 | 16.32 | 392.5 |
| 6 | Pool-6 | 4.31 | 186.75 |
| 7 | Pool-7 | 1.84 | 78.75 |
| 8 | Pool-8 | 0 | 0 |

Average standard concentration = 0.375 µg

Average standard height = 19.4325 mm

**Table: Determination of Meningococcal Polysaccharide content (Group-Y)**

| **Well No.** | **Name of Standard** | **Rocket Height (mm )** | ***Concentration (µg)** |
| --- | --- | --- | --- |
| 1 | Std-0.5 | 7.12 | 0.05 |
| 2 | Std-1 | 9.33 | 0.1 |
| 3 | Std-2 | 11.47 | 0.2 |
| 4 | Std-4 | 13.56 | 0.4 |
| 5 | Std-8 | 15.98 | 0.8 |
|  | | | |
| **Sl. NO.** | **Sample Pool No.** | **Rocket Height (mm )** | **Total Polysaccharide, µg** |
| 1 | Pool-1 | 0 | 0 |
| 2 | Pool-2 | 9.97 | 335 |
| 3 | Pool-3 | 12.78 | 430 |
| 4 | Pool-4 | 13.81 | 465 |
| 5 | Pool-5 | 13.02 | 438.75 |
| 6 | Pool-6 | 2.14 | 85.5 |
| 7 | Pool-7 | 0.89 | 54 |
| 8 | Pool-8 | 0 | 0 |
| 9 | Pool-9 | 0 | 0 |

Average standard concentration = 0.31 µg

Average standard height = 11.492 mm

**Batch: 15004**

**Table: Determination of Meningococcal Polysaccharide content (Group-A )**

| **Well No.** | **Name of Std** | **Rocket Height (mm )** | ***Concentration (µg)** |
| --- | --- | --- | --- |
| 1 | Std-0.5 | 16.52 | 0.05 |
| 2 | Std-1 | 17.99 | 0.1 |
| 3 | Std-2 | 19.29 | 0.2 |
| 4 | Std-4 | 22.04 | 0.4 |
| 5 | Std-8 | 25.76 | 0.8 |
|  | | | |
| **Sl. NO.** | **Sample Pool No.** | **Rocket Height (mm )** | **Total Polysaccharide, µg** |
| 1 | Pool-1 | 0 | 0 |
| 2 | Pool-2 | 21.92 | 417.5 |
| 3 | Pool-3 | 18.51 | 282 |
| 4 | Pool-4 | 16.96 | 388.5 |
| 5 | Pool-5 | 15.73 | 298.75 |
| 6 | Pool-6 | 7.55 | 172.5 |
| 7 | Pool-7 | 3.94 | 135 |
| 8 | Pool-8 | 0 | 0 |
| 9 | Pool-9 | 0 | 0 |

**Calculation:**

Average standard concentration = 0.31 µg

Average standard height = 20.32 mm

**Table: Determination of Meningococcal Polysaccharide content (Group-C )**

| **Well No.** | **Name of Standard** | **Rocket Height (mm )** | ***Concentration (µg)** |
| --- | --- | --- | --- |
| 1 | Std-0.5 | 15.70 | 0.05 |
| 2 | Std-1 | 18.83 | 0.1 |
| 3 | Std-2 | 21.21 | 0.2 |
| 4 | Std-4 | 25.85 | 0.4 |
| 5 | Std-8 | 28.29 | 0.8 |
|  | | | |
| **Sl. NO.** | **Sample Pool No.** | **Rocket Height (mm )** | **Total Polysaccharide, µg** |
| 1 | Pool-1 | 0 | 0 |
| 2 | Pool-2 | 27.61 | 486.25 |
| 3 | Pool-3 | 21.88 | 309 |
| 4 | Pool-4 | 14.62 | 309 |
| 5 | Pool-5 | 13.52 | 238.75 |
| 6 | Pool-6 | 8.26 | 175.5 |
| 7 | Pool-7 | 2.35 | 74.25 |
| 8 | Pool-8 | 1.01 | 31.5 |
| 9 | Pool-9 | 0 | 0 |

**Calculation:**

Average standard concentration = 0.31 µg

Average standard height = 21.976 mm

**Table: Determination of Meningococcal Polysaccharide content (Group-W135)**

| **Well No.** | **Name of Standard** | **Rocket Height (mm )** | ***Concentration (µg)** |
| --- | --- | --- | --- |
| 1 | Std-1 | 17.96 | 0.1 |
| 2 | Std-2 | 21.61 | 0.2 |
| 3 | Std-4 | 24.21 | 0.4 |
| 4 | Std-8 | 26.96 | 0.8 |
|  | | | |
| **Sl. NO.** | **Sample Pool No.** | **Rocket Height (mm )** | **Total Polysaccharide, µg** |
| 1 | Pool-1 | 0 | 0 |
| 2 | Pool-2 | 25.54 | 527.5 |
| 3 | Pool-3 | 19.99 | 330 |
| 4 | Pool-4 | 14.32 | 355.5 |
| 5 | Pool-5 | 12.86 | 265 |
| 6 | Pool-6 | 5.64 | 135 |
| 7 | Pool-7 | 1.58 | 58.5 |
| 8 | Pool-8 | 0 | 0 |

Average standard concentration = 0.375 µg

Average standard height = 22.685 mm

**Table: Determination of Meningococcal Polysaccharide content (Group-Y)**

| **Well No.** | **Name of Standard** | **Rocket Height (mm )** | ***Concentration (µg)** |
| --- | --- | --- | --- |
| 1 | Std-1 | 10.78 | 0.1 |
| 2 | Std-2 | 12.69 | 0.2 |
| 3 | Std-4 | 14.93 | 0.4 |
| 4 | Std-8 | 17.08 | 0.8 |
|  | | | |
| **Sl. NO.** | **Sample Pool No.** | **Rocket Height (mm )** | **Total Polysaccharide, µg** |
| 1 | Pool-1 | 0 | 0 |
| 2 | Pool-2 | 14.99 | 506.25 |
| 3 | Pool-3 | 12.01 | 325 |
| 4 | Pool-4 | 11.87 | 481.5 |
| 5 | Pool-5 | 8.17 | 276.25 |
| 6 | Pool-6 | 2.43 | 97.5 |
| 7 | Pool-7 | 0.91 | 56.25 |
| 8 | Pool-8 | 0 | 0 |

**Calculation:**

Average standard concentration = 0.375 µg

Average standard height = 13.87mm

**Batch: 15005**

**Table: Determination of Meningococcal Polysaccharide content (Group-A )**

| **Well No.** | **Name of Standard** | **Rocket Height (mm )** | ***Concentration (µg)** |
| --- | --- | --- | --- |
| 1 | Std-0.5 | 12.87 | 0.05 |
| 2 | Std-1 | 13.99 | 0.1 |
| 3 | Std-2 | 15.76 | 0.2 |
| 4 | Std-4 | 17.43 | 0.4 |
| 5 | Std-8 | 19.01 | 0.8 |
|  | | | |
| **Sl. NO.** | **Sample Pool No.** | **Rocket Height (mm )** | **Total Polysaccharide, µg** |
| 1 | Pool-1 | 0 | 0 |
| 2 | Pool-2 | 13.39 | 326.25 |
| 3 | Pool-3 | 14.01 | 324 |
| 4 | Pool-4 | 15.42 | 652.75 |
| 5 | Pool-5 | 9.90 | 185 |
| 6 | Pool-6 | 6.98 | 178.5 |
| 7 | Pool-7 | 3.08 | 37.5 |
| 8 | Pool-8 | 0 | 0 |
| 9 | Pool-9 | 0 | 0 |

Average standard concentration = 0.31 µg

Average standard height = 15.812 mm

**Table: Determination of Meningococcal Polysaccharide content (Group-C )**

| **Well No.** | **Name of Standard** | **Rocket Height (mm )** | ***Concentration (µg)** |
| --- | --- | --- | --- |
| 1 | Std-0.5 | 8.88 | 0.05 |
| 2 | Std-1 | 9.06 | 0.1 |
| 3 | Std-2 | 11.01 | 0.2 |
| 4 | Std-4 | 12.52 | 0.4 |
| 5 | Std-8 | 14.07 | 0.8 |
|  | | | |
| **Sl. NO.** | **Sample Pool No.** | **Rocket Height (mm )** | **Total Polysaccharide, µg** |
| 1 | Pool-1 | 0 | 0 |
| 2 | Pool-2 | 9.06 | 315 |
| 3 | Pool-3 | 10.41 | 291 |
| 4 | Pool-4 | 12.39 | 605.5 |
| 5 | Pool-5 | 8.92 | 311.25 |
| 6 | Pool-6 | 4.01 | 168 |
| 7 | Pool-7 | 1.96 | 135 |
| 8 | Pool-8 | 0 | 0 |
| 9 | Pool-9 | 0 | 0 |

Average standard concentration = 0.31 µg

Average standard height = 11.108 mm

**Table: Determination of Meningococcal Polysaccharide content (Group-W135)**

| **Well No.** | **Name of Standard** | **Rocket Height (mm )** | ***Concentration (µg)** |
| --- | --- | --- | --- |
| 1 | Std-1 | 15.98 | 0.1 |
| 2 | Std-2 | 18.13 | 0.2 |
| 3 | Std-4 | 21.21 | 0.4 |
| 4 | Std-8 | 24.05 | 0.8 |
|  | | | |
| **Sl. NO.** | **Sample Pool No.** | **Rocket Height (mm )** | **Total Polysaccharide, µg** |
| 1 | Pool-1 | 0 | 0 |
| 2 | Pool-2 | 13.80 | 326.25 |
| 3 | Pool-3 | 17.18 | 324 |
| 4 | Pool-4 | 19.76 | 652.75 |
| 5 | Pool-5 | 7.85 | 185 |
| 6 | Pool-6 | 6.29 | 178.5 |
| 7 | Pool-7 | 0.81 | 37.5 |
| 8 | Pool-8 | 0 | 0 |

Average standard concentration = 0.375 µg

Average standard height = 19.843 mm

Table**: Determination of Meningococcal Polysaccharide content (Group-Y)**

| **Well No.** | **Name of Standard** | **Rocket Height (mm )** | ***Concentration (µg)** |
| --- | --- | --- | --- |
| 1 | Std-1 | 8.14 | 0.1 |
| 2 | Std-2 | 9.52 | 0.2 |
| 3 | Std-4 | 10.98 | 0.4 |
| 4 | Std-8 | 12.21 | 0.8 |
|  | | | |
| **Sl. NO.** | **Sample Pool No.** | **Rocket Height (mm )** | **Total Polysaccharide, µg** |
| 1 | Pool-1 | 0 | 0 |
| 2 | Pool-2 | 10.19 | 467.5 |
| 3 | Pool-3 | 11.00 | 404 |
| 4 | Pool-4 | 7.43 | 477.75 |
| 5 | Pool-5 | 5.23 | 240 |
| 6 | Pool-6 | 2.98 | 163.5 |
| 7 | Pool-7 | 0.06 | 5 |
| 8 | Pool-8 | 0 | 0 |

Average standard concentration = 0.375 µg

Average standard height = 10.213 mm

**Batch: 15006**

**Table: Determination of Meningococcal Polysaccharide content (Group-A )**

| **Well No.** | **Name of Standard** | **Rocket Height (mm )** | ***Concentration (µg)** |
| --- | --- | --- | --- |
| 1 | Std-0.5 | 7.91 | 0.05 |
| 2 | Std-1 | 10.01 | 0.1 |
| 3 | Std-2 | 11.97 | 0.2 |
| 4 | Std-4 | 13.76 | 0.4 |
| 5 | Std-8 | 16.10 | 0.8 |
|  | | | |
| **Sl. NO.** | **Sample Pool No.** | **Rocket Height (mm )** | **Total Polysaccharide, µg** |
| 1 | Pool-1 | 0 | 0 |
| 2 | Pool-2 | 10.4 | 336.25 |
| 3 | Pool-3 | 13.51 | 350 |
| 4 | Pool-4 | 12.07 | 547.75 |
| 5 | Pool-5 | 9.71 | 315 |
| 6 | Pool-6 | 4.15 | 162 |
| 7 | Pool-7 | 0.89 | 57.5 |
| 8 | Pool-8 | 0 | 0 |
| 9 | Pool-9 | 0 | 0 |

Average standard concentration = 0.31 µg

Average standard height = 11.95 mm

**Table: Determination of Meningococcal Polysaccharide content (Group-C )**

| **Well No.** | **Name of Standard** | **Rocket Height (mm )** | ***Concentration (µg)** |
| --- | --- | --- | --- |
| 1 | Std-0.5 | 13.81 | 0.05 |
| 2 | Std-1 | 15.65 | 0.1 |
| 3 | Std-2 | 16.42 | 0.2 |
| 4 | Std-4 | 18.63 | 0.4 |
| 5 | Std-8 | 19.69 | 0.8 |
|  | | | |
| **Sl. NO.** | **Sample Pool No.** | **Rocket Height (mm )** | **Total Polysaccharide, µg** |
| 1 | Pool-1 | 0 | 0 |
| 2 | Pool-2 | 16.41 | 377.5 |
| 3 | Pool-3 | 17.02 | 313 |
| 4 | Pool-4 | 15.19 | 488.25 |
| 5 | Pool-5 | 8.41 | 198.75 |
| 6 | Pool-6 | 6.91 | 190.5 |
| 7 | Pool-7 | 2.09 | 95 |
| 8 | Pool-8 | 0 | 0 |
| 9 | Pool-9 | 0 | 0 |

Average standard concentration = 0.31 µg

Average standard height = 16.84 mm

**Table: Determination of Meningococcal Polysaccharide content (Group-W135)**

| **Well No.** | **Name of Standard** | **Rocket Height (mm )** | ***Concentration (µg)** |
| --- | --- | --- | --- |
| 1 | Std-1 | 11.60 | 0.1 |
| 2 | Std-2 | 13.19 | 0.2 |
| 3 | Std-4 | 15.36 | 0.4 |
| 4 | Std-8 | 17.08 | 0.8 |
|  | | | |
| **Sl. NO.** | **Sample Pool No.** | **Rocket Height (mm )** | **Total Polysaccharide, µg** |
| 1 | Pool-1 | 0 | 0 |
| 2 | Pool-2 | 13.23 | 433.75 |
| 3 | Pool-3 | 14.08 | 369 |
| 4 | Pool-4 | 10.21 | 469 |
| 5 | Pool-5 | 7.81 | 256.25 |
| 6 | Pool-6 | 5.94 | 234 |
| 7 | Pool-7 | 0.81 | 52.5 |
| 8 | Pool-8 | 0 | 0 |

Average standard concentration = 0.375 µg

Average standard height = 14.31 mm

**Table: Determination of Meningococcal Polysaccharide content (Group-Y)**

| **Well No.** | **Name of Standard** | **Rocket Height (mm )** | ***Concentration (µg)** |
| --- | --- | --- | --- |
| 1 | Std-1 | 16.81 | 0.1 |
| 2 | Std-2 | 17.97 | 0.2 |
| 3 | Std-4 | 19.29 | 0.4 |
| 4 | Std-8 | 21.03 | 0.8 |
|  | | | |
| **Sl. NO.** | **Sample Pool No.** | **Rocket Height (mm )** | **Total Polysaccharide, µg** |
| 1 | Pool-1 | 0 | 0 |
| 2 | Pool-2 | 17.20 | 428.75 |
| 3 | Pool-3 | 19.61 | 392 |
| 4 | Pool-4 | 14.32 | 500.5 |
| 5 | Pool-5 | 9.41 | 233.75 |
| 6 | Pool-6 | 5.18 | 154.5 |
| 7 | Pool-7 | 2.19 | 110 |
| 8 | Pool-8 | 0 | 0 |

Average standard concentration = 0.375 µg

Average standard height = 18.775 mm
