# Supplementary material for: A High-Throughput Size Exclusion Chromatography Method to Determine the Molecular Size Distribution of Meningococcal Polysaccharide Vaccine
Source: Int J Anal Chem. 2016 Sep 5;2016:9404068. doi: 10.1155/2016/9404068 (PMC5027369; doi:10.1155/2016/9404068)
Supplement: Supplementary file 1 — S1: The polysaccharide content of all fractions was calculated from the value of rocket height of samples & standards and standard's concentrations. S2: The total percentage of recovered polysaccharide for all serogroups was shown. [file 9404068.f1.zip › S2.docx]

**S2: Molecular size distribution analyses of six batches of Ingovax^®^ACWY polysaccharide content in eluted fractions**

**Batch: 15001**

**Group A:**

| **Pool Number** | **Pool Volume, mL** | **Polysaccharide, µg/8µl** | **Fractions Included** | | | | | | **Polysaccharide, µg/mL** | | **Total Polysaccharide, µg** |
| --- | --- | --- | --- | --- | --- | --- | --- | --- | --- | --- | --- |
| Pool 1 | 28 | 0 | A1+A2+A3+A4+A5+A6+A7+A8+A9+A10+A11+A12+B1+B2 | | | | | | 0 | | 0 |
| Pool 2 | 10 | 0.2114 | B3+B4+B5+B6+B7 | | | | | | 26.425 | | 264.25 |
| Pool 3 | 10 | 0.263 | B8+B9+B10+B11+B12 | | | | | | 32.875 | | 328.75 |
| Pool 4 | 10 | 0.3169 | C1+C2+C3+C4+C5 | | | | | | 39.6125 | | 396.125 |
| Pool 5 | 10 | 0.3514 | C6+C7+C8+C9+C10 | | | | | | 43.925 | | 439.25 |
| Pool 6 | 12 | 0.176 | C11+C12+D1+D2+D3+D4 | | | | | | 22 | | 264 |
| Pool 7 | 18 | 0.088 | D5+D6+D7+D8+D9+D10+D11+D12+E1 | | | | | | 11 | | 198 |
| Pool 8 | 20 | 0 | E2+E3+E4+E5+E6+E7+E8+E9+E10+E11 | | | | | | 0 | | 0 |
| Pool 9 | 26 | 0 | E12+F1+F2+F3+F4+F5+F6+F7+F8+F9+F10+F11 +F12 | | | | | | 0 | | 0 |
|  |  |  | **Total Recovered Polysaccharide** | | | | | | | | **1890.375** |
| Polysaccharide Group | Polysaccharide Content/Vial, µg | Polysaccharide Content in 40 Vials, µg | Polysaccharide Content Loaded, µg | Recovered polysaccharide in elution before Kd 0.5, µg | | | | | *Recovered polysaccharide in elution before Kd 0.5, % | | Complies BP Requirement |
| A | 62.475 | 2499.00 | 1999.20 | 1890.38 | | | | | 94.56 | | Yes |
| **Group C:** | | | | | | | | | | | |
| **Pool Number** | **Pool Volume, mL** | **Polysaccharide, µg/8µl** | **Fractions Included** | | | | | | **Polysaccharide, µg/mL** | | **Total Polysaccharide, µg** |
| Pool 1 | 28 | 0 | A1+A2+A3+A4+A5+A6+A7+A8+A9+A10+A11+A12+B1+B2 | | | | | | 0 | | 0 |
| Pool 2 | 10 | 0.3048 | B3+B4+B5+B6+B7 | | | | | | 38.1 | | 381 |
| Pool 3 | 10 | 0.3059 | B8+B9+B10+B11+B12 | | | | | | 38.2375 | | 382.375 |
| Pool 4 | 10 | 0.3921 | C1+C2+C3+C4+C5 | | | | | | 49.0125 | | 490.125 |
| Pool 5 | 10 | 0.3045 | C6+C7+C8+C9+C10 | | | | | | 38.0625 | | 380.625 |
| Pool 6 | 12 | 0.1552 | C11+C12+D1+D2+D3+D4 | | | | | | 19.4 | | 232.8 |
| Pool 7 | 18 | 0 | D5+D6+D7+D8+D9+D10+D11+D12+E1 | | | | | | 0 | | 0 |
| Pool 8 | 46 | 0 | E2+E3+E4+E5+E6+E7+E8+E9+E10+E11+ E12+ F1+ F2+ F3+ +F4 +F5+ F6+ F7+ F8+ F9+ F10+F11+F12 | | | | | | 0 | | 0 |
|  |  |  | **Total Recovered Polysaccharide** | | | | | | | | **1866.925** |
| Polysaccharide Group | Polysaccharide Content/Vial, µg | Polysaccharide Content in 40 Vials, µg | Polysaccharide Content Loaded, µg | | Recovered polysaccharide in elution before Kd 0.5, µg | | | | *Recovered polysaccharide in elution before Kd 0.5, % | | Complies BP Requirement |
| C | 62.37 | 2494.80 | 1918.50 | | 1866.93 | | | | 97.31 | | Yes |
| **Group W:** | | | | | | | | | | | |
| **Pool Number** | **Pool Volume, mL** | **Polysaccharide, µg/8µl** | **Fractions Included** | | | | | | **Polysaccharide, µg/mL** | | **Total Polysaccharide, µg** |
| Pool 1 | 28 | 0 | A1+A2+A3+A4+A5+A6+A7+A8+A9+A10+A11+A12+ B1+B2 | | | | | | 0 | | 0 |
| Pool 2 | 10 | 0.3107 | B3+B4+B5+B6+B7 | | | | | | 38.8375 | | 388.375 |
| Pool 3 | 10 | 0.2981 | B8+B9+B10+B11+B12 | | | | | | 37.2625 | | 372.625 |
| Pool 4 | 10 | 0.3126 | C1+C2+C3+C4+C5 | | | | | | 39.075 | | 390.75 |
| Pool 5 | 10 | 0.2955 | C6+C7+C8+C9+C10 | | | | | | 36.9375 | | 369.375 |
| Pool 6 | 12 | 0.1205 | C11+C12+D1+D2+D3+D4 | | | | | | 15.0625 | | 180.75 |
| Pool 7 | 18 | 0.0159 | D5+D6+D7+D8+D9+D10+D11+D12+E1 | | | | | | 1.9875 | | 35.775 |
| Pool 8 | 46 | 0 | E2+E3+E4+E5+E6+E7+E8+E9+E10+E11+ E12+ F1+ F2+ F3+F4+F5+F6+F7+F8+F9+F10+F11+F12 | | | | | | 0 | | 0 |
|  |  |  | **Total Recovered Polysaccharide** | | | | | | | | **1737.65** |
| Polysaccharide Group | Polysaccharide Content/Vial, µg | Polysaccharide Content in 40 Vials, µg | Polysaccharide Content Loaded, µg | Recovered polysaccharide in elution before Kd 0.5, µg | | | | *Recovered polysaccharide in elution before Kd 0.5, % | | | Complies BP Requirement |
| W | 68.1785 | 2727.14 | 2097.17 | 1701.88 | | | | 81.15 | | | Yes |
| **Group Y:** | | | | | | | | | | | |
| **Pool Number** | **Pool Volume, mL** | **Polysaccharide, µg/8µl** | **Fractions Included** | | | | | **Polysaccharide, µg/mL** | | | **Total Polysaccharide, µg** |
| Pool 1 | 28 | 0 | A1+A2+A3+A4+A5+A6+A7+A8+A9+A10+A11+A12+B1+B2 | | | | | 0 | | | 0 |
| Pool 2 | 10 | 0.351 | B3+B4+B5+B6+B7 | | | | | 43.875 | | | 438.75 |
| Pool 3 | 10 | 0.3253 | B8+B9+B10+B11+B12 | | | | | 40.6625 | | | 406.625 |
| Pool 4 | 10 | 0.3131 | C1+C2+C3+C4+C5 | | | | | 39.1375 | | | 391.375 |
| Pool 5 | 10 | 0.2718 | C6+C7+C8+C9+C10 | | | | | 33.975 | | | 339.75 |
| Pool 6 | 12 | 0.0969 | C11+C12+D1+D2+D3+D4 | | | | | 12.1125 | | | 145.35 |
| Pool 7 | 18 | 0.0149 | D5+D6+D7+D8+D9+D10+D11+D12+E1 | | | | | 1.8625 | | | 33.525 |
| Pool 8 | 20 | 0 | E2+E3+E4+E5+E6+E7+E8+E9+E10+E11 | | | | | 0 | | | 0 |
| Pool 9 | 26 | 0 | E12+F1+F2+F3+F4+F5+F6+F7+F8+F9+F10+F11+ F12 | | | | | 0 | | | 0 |
|  |  |  | **Total Recovered Polysaccharide** | | | | | | | | **1755.375** |
| Polysaccharide Group | Polysaccharide Content/Vial, µg | Polysaccharide Content in 40 Vials, µg | Polysaccharide Content Loaded, µg | | Recovered polysaccharide in elution before Kd 0.5, µg | | | | *Recovered polysaccharide in elution before Kd 0.5, % | | Complies BP Requirement |
| Y | 68.969 | 2758.76 | 2121.49 | | 1721.85 | | | | 81.16 | | Yes |
| **Batch: 15002**  **Group A:** | | | | | | | | | | | |
| **Pool Number** | **Pool Volume, mL** | **Polysaccharide, µg/8µl** | **Fractions Included** | | | | | **Polysaccharide, µg/mL** | | | **Total Polysaccharide, µg** |
| Pool 1 | 26 | 0 | A1+A2+A3+A4+A5+A6+A7+A8+A9+A10+A11+A12+B1 | | | | | 0 | | | 0 |
| Pool 2 | 10 | 0.225 | B2+B3+B4+B5+B6 | | | | | 28.125 | | | 281.25 |
| Pool 3 | 14 | 0.2374 | B7+B8+B9+B10+B11+B12+C1 | | | | | 29.675 | | | 415.45 |
| Pool 4 | 12 | 0.273 | C2+C3+C4+C5+C6+C7 | | | | | 34.125 | | | 409.5 |
| Pool 5 | 12 | 0.3013 | C8+C9+C10+C11+C12+D1 | | | | | 37.6625 | | | 451.95 |
| Pool 6 | 10 | 0.153 | D2+D3+D4+D5+D6 | | | | | 19.125 | | | 191.25 |
| Pool 7 | 14 | 0.0814 | D7+D8+D9+D10+D11+D12+E1 | | | | | 10.175 | | | 142.45 |
| Pool 8 | 20 | 0 | E2+E3+E4+E5+E6+E7+E8+E9+E10+E11 | | | | | 0 | | | 0 |
| Pool 9 | 26 | 0 | E12+F1+F2+F3+F4+F5+F6+F7+F8+F9+F10+F11+F12 | | | | | 0 | | | 0 |
|  |  |  | **Total Recovered Polysaccharide** | | | | | | | | **1891.85** |
| Polysaccharide Group | Polysaccharide Content/Vial, µg | Polysaccharide Content in 40 Vials, µg | Polysaccharide Content Loaded, µg | | | Recovered polysaccharide in elution before Kd 0.5, µg | *Recovered polysaccharide in elution before Kd 0.5, % | | | | Complies BP Requirement |
| A | 59.984 | 2399.36 | 1919.49 | | | 1891.85 | 98.56 | | | | Yes |
| **Group C:** | | | | | | | | | | | |
| **Pool Number** | **Pool Volume, mL** | **Polysaccharide, µg/8µl** | **Fractions Included** | | | | **Polysaccharide, µg/mL** | | | **Total Polysaccharide, µg** | |
| Pool 1 | 26 | 0 | A1+A2+A3+A4+A5+A6+A7+A8+A9+A10+A11+A12+B1 | | | | 0 | | | 0 | |
| Pool 2 | 10 | 0.235 | B2+B3+B4+B5+B6 | | | | 29.375 | | | 293.75 | |
| Pool 3 | 14 | 0.312 | B7+B8+B9+B10+B11+B12+C1 | | | | 39 | | | 546 | |
| Pool 4 | 12 | 0.379 | C2+C3+C4+C5+C6+C7 | | | | 47.375 | | | 568.5 | |
| Pool 5 | 12 | 0.29 | C8+C9+C10+C11+C12+D1 | | | | 36.25 | | | 435 | |
| Pool 6 | 10 | 0.09 | D2+D3+D4+D5+D6 | | | | 11.25 | | | 112.5 | |
| Pool 7 | 14 | 0.029 | D7+D8+D9+D10+D11+D12+E1 | | | | 3.625 | | | 50.75 | |
| Pool 8 | 46 | 0 | E12+F1+F2+F3+F4+F5+F6+F7+F8+F9+F10+F11+F12 | | | | 0 | | | 0 | |
|  |  |  | **Total Recovered Polysaccharide** | | | | | | | **2006.5** | |
| Polysaccharide Group | Polysaccharide Content/Vial, µg | Polysaccharide Content in 40 Vials, µg | Polysaccharide Content Loaded, µg | | | Recovered polysaccharide in elution before Kd 0.5, µg | *Recovered polysaccharide in elution before Kd 0.5, % | | | Complies BP Requirement | |
| C | 64.599 | 2583.96 | 2067.17 | | | 2006.5 | 97.07 | | | Yes | |
|  | | | | | | | | | | | |
| **Group W:** | | | | | | | | | | | |
| **Pool Number** | **Pool Volume, mL** | **Polysaccharide, µg/8µl** | **Fractions Included** | | | | **Polysaccharide, µg/mL** | | | **Total Polysaccharide, µg** | |
| Pool 1 | 26 | 0 | A1+A2+A3+A4+A5+A6+A7+A8+A9+A10+A11+A12+B1 | | | | 0 | | | 0 | |
| Pool 2 | 10 | 0.294 | B2+B3+B4+B5+B6 | | | | 36.75 | | | 367.5 | |
| Pool 3 | 14 | 0.312 | B7+B8+B9+B10+B11+B12+C1 | | | | 39 | | | 546 | |
| Pool 4 | 12 | 0.355 | C2+C3+C4+C5+C6+C7 | | | | 44.375 | | | 532.5 | |
| Pool 5 | 12 | 0.271 | C8+C9+C10+C11+C12+D1 | | | | 33.875 | | | 406.5 | |
| Pool 6 | 10 | 0.0745 | D2+D3+D4+D5+D6 | | | | 9.3125 | | | 93.125 | |
| Pool 7 | 14 | 0.0179 | D7+D8+D9+D10+D11+D12+E1 | | | | 2.2375 | | | 31.325 | |
| Pool 8 | 46 | 0 | E12+F1+F2+F3+F4+F5+F6+F7+F8+F9+F10+F11+F12 | | | | 0 | | | 0 | |
|  |  |  | **Total Recovered Polysaccharide** | | | | | | | **1976.95** | |
| Polysaccharide Group | Polysaccharide Content/Vial, µg | Polysaccharide Content in 40 Vials, µg | Polysaccharide Content Loaded, µg | | | Recovered polysaccharide in elution before Kd 0.5, µg | *Recovered polysaccharide in elution before Kd 0.5, % | | | Complies BP Requirement | |
| W | 64.201 | 2568.04 | 2054.43 | | | 1976.95 | 96.23 | | | Yes | |
| **Group Y:** | | | | | | | | | | | |
| **Pool Number** | **Pool Volume, mL** | **Polysaccharide, µg/8µl** | **Fractions Included** | | | | **Polysaccharide, µg/mL** | | | **Total Polysaccharide, µg** | |
| Pool 1 | 26 | 0 | A1+A2+A3+A4+A5+A6+A7+A8+A9+A10+A11+A12+B1 | | | | 0 | | | 0 | |
| Pool 2 | 10 | 0.167 | B2+B3+B4+B5+B6 | | | | 20.875 | | | 208.75 | |
| Pool 3 | 14 | 0.307 | B7+B8+B9+B10+B11+B12+C1 | | | | 38.375 | | | 537.25 | |
| Pool 4 | 12 | 0.383 | C2+C3+C4+C5+C6+C7 | | | | 47.875 | | | 574.5 | |
| Pool 5 | 12 | 0.26 | C8+C9+C10+C11+C12+D1 | | | | 32.5 | | | 390 | |
| Pool 6 | 10 | 0.1109 | D2+D3+D4+D5+D6 | | | | 13.8625 | | | 138.625 | |
| Pool 7 | 14 | 0.0238 | D7+D8+D9+D10+D11+D12+E1 | | | | 2.975 | | | 41.65 | |
| Pool 8 | 20 | 0 | E2+E3+E4+E5+E6+E7+E8+E9+E10+E11 | | | | 0 | | | 0 | |
| Pool 9 | 26 | 0 | E12+F1+F2+F3+F4+F5+F6+F7+F8+F9+F10+F11+F12 | | | | 0 | | | 0 | |
|  |  |  | **Total Recovered Polysaccharide** | | | | | | | **1890.775** | |
| Polysaccharide Group | Polysaccharide Content/Vial, µg | Polysaccharide Content in 40 Vials, µg | Polysaccharide Content Loaded, µg | | | Recovered polysaccharide in elution before Kd 0.5, µg | *Recovered polysaccharide in elution before Kd 0.5, % | | | Complies BP Requirement | |
| Y | 69.67 | 2786.80 | 2229.44 | | | 1890.775 | 84.81 | | | Yes | |

| **Batch: 15003**  **Group A:** | | | | | | | | | | | |
| --- | --- | --- | --- | --- | --- | --- | --- | --- | --- | --- | --- |
| **Pool Number** | **Pool Volume, mL** | **Polysaccharide, µg/8µl** | **Fractions Included** | | | | | | **Polysaccharide, µg/mL** | | **Total Polysaccharide, µg** |
| Pool 1 | 28 | 0 | A1+A2+A3+A4+A5+A6+A7+A8+A9+A10+A11+A12+B1+B2 | | | | | | 0 | | 0 |
| Pool 2 | 10 | 0.232 | B3+B4+B5+B6+B7 | | | | | | 29 | | 290 |
| Pool 3 | 10 | 0.238 | B8+B9+B10+B11+B12 | | | | | | 29.75 | | 297.5 |
| Pool 4 | 10 | 0.27 | C1+C2+C3+C4+C5 | | | | | | 33.75 | | 337.5 |
| Pool 5 | 10 | 0.305 | C6+C7+C8+C9+C10 | | | | | | 38.125 | | 381.25 |
| Pool 6 | 12 | 0.264 | C11+C12+D1+D2+D3+D4 | | | | | | 33 | | 396 |
| Pool 7 | 18 | 0.096 | D5+D6+D7+D8+D9+D10+D11+D12+E1 | | | | | | 12 | | 216 |
| Pool 8 | 18 | 0 | E2+E3+E4+E5+E6+E7+E8+E9+E10 | | | | | | 0 | | 0 |
| Pool 9 | 28 | 0 | E11+E12+F1+F2+F3+F4+F5+F6+F7+F8+F9+F10+F11+F12 | | | | | | 0 | | 0 |
|  |  |  | **Total Recovered Polysaccharide** | | | | | | | | **1918.25** |
|  |  |  |  | | |  | |  | | |  |
| Polysaccharide Group | Polysaccharide Content/Vial, µg | Polysaccharide Content in 40 Vials, µg | Polysaccharide Content Loaded, µg | | | Recovered polysaccharide in elution before Kd 0.5, µg | | *Recovered polysaccharide in elution before Kd 0.5, % | | | Complies BP Requirement |
| A | 61.72 | 2468.80 | 1975.04 | | | 1918.25 | | 97.12 | | | Yes |
| **Group C:** | | | | | | | | | | | |
| **Pool Number** | **Pool Volume, mL** | **Polysaccharide, µg/8µl** | **Fractions Included** | | | | | **Polysaccharide, µg/mL** | | **Total Polysaccharide, µg** | |
| Pool 1 | 28 | 0 | A1+A2+A3+A4+A5+A6+A7+A8+A9+A10+A11+A12+B1+B2 | | | | | 0 | | 0 | |
| Pool 2 | 10 | 0.301 | B3+B4+B5+B6+B7 | | | | | 37.625 | | 376.25 | |
| Pool 3 | 10 | 0.34 | B8+B9+B10+B11+B12 | | | | | 42.5 | | 425 | |
| Pool 4 | 10 | 0.376 | C1+C2+C3+C4+C5 | | | | | 47 | | 470 | |
| Pool 5 | 10 | 0.32 | C6+C7+C8+C9+C10 | | | | | 40 | | 400 | |
| Pool 6 | 12 | 0.127 | C11+C12+D1+D2+D3+D4 | | | | | 15.875 | | 190.5 | |
| Pool 7 | 18 | 0.027 | D5+D6+D7+D8+D9+D10+D11+D12+E1 | | | | | 3.375 | | 60.75 | |
| Pool 8 | 46 | 0 | E2+E3+E4+E5+E6+E7+E8+E9+E10+E11+E12+F1+F2+ F3+F4+F5+F6+F7+F8+F9+F10+F11+F12 | | | | | 0 | | 0 | |
|  |  |  | **Total Recovered Polysaccharide** | | | | | | | **1922.5** | |
| Polysaccharide Group | Polysaccharide Content/Vial, µg | Polysaccharide Content in 40 Vials, µg | Polysaccharide Content Loaded, µg | | Recovered polysaccharide in elution before Kd 0.5, µg | | *Recovered polysaccharide in elution before Kd 0.5, % | | | Complies BP Requirement | |
| C | 67.621 | 2704.84 | 2163.87 | | 1922.5 | | 88.85 | | | Yes | |
| **Group W:** | | | | | | | | | | | |
| **Pool Number** | **Pool Volume, mL** | **Polysaccharide, µg/8µl** | **Fractions Included** | | | | **Polysaccharide, µg/mL** | | | **Total Polysaccharide, µg** | |
| Pool 1 | 28 | 0 | A1+A2+A3+A4+A5+A6+A7+A8+A9+A10+A11+A12+B1+B2 | | | | 0 | | | 0 | |
| Pool 2 | 10 | 0.305 | B3+B4+B5+B6+B7 | | | | 38.125 | | | 381.25 | |
| Pool 3 | 10 | 0.346 | B8+B9+B10+B11+B12 | | | | 43.25 | | | 432.5 | |
| Pool 4 | 10 | 0.348 | C1+C2+C3+C4+C5 | | | | 43.5 | | | 435 | |
| Pool 5 | 10 | 0.314 | C6+C7+C8+C9+C10 | | | | 39.25 | | | 392.5 | |
| Pool 6 | 12 | 0.083 | C11+C12+D1+D2+D3+D4 | | | | 10.375 | | | 124.5 | |
| Pool 7 | 18 | 0.035 | D5+D6+D7+D8+D9+D10+D11+D12+E1 | | | | 4.375 | | | 78.75 | |
| Pool 8 | 46 | 0 | E2+E3+E4+E5+E6+E7+E8+E9+E10+E11+E12+F1+F2+F3+F4+F5+F6+F7+F8+F9+F10+F11+F12 | | | | 0 | | | 0 | |
|  |  |  | **Total Recovered Polysaccharide** | | | | | | | **1844.5** | |
| Polysaccharide Group | Polysaccharide Content/Vial, µg | Polysaccharide Content in 40 Vials, µg | Polysaccharide Content Loaded, µg | Recovered polysaccharide in elution before Kd 0.5, µg | | | *Recovered polysaccharide in elution before Kd 0.5, % | | | Complies BP Requirement | |
| W | 62.506 | 2500.24 | 2000.19 | 1844.5 | | | 92.22 | | | Yes | |
| **Group Y:** | | | | | | | | | | | |
| **Pool Number** | **Pool Volume, mL** | **Polysaccharide, µg/8µl** | **Fractions Included** | | | | **Polysaccharide, µg/mL** | | | **Total Polysaccharide, µg** | |
| Pool 1 | 28 | 0 | A1+A2+A3+A4+A5+A6+A7+A8+A9+A10+A11+A12+B1+B2 | | | | 0 | | | 0 | |
| Pool 2 | 10 | 0.268 | B3+B4+B5+B6+B7 | | | | 33.5 | | | 335 | |
| Pool 3 | 10 | 0.344 | B8+B9+B10+B11+B12 | | | | 43 | | | 430 | |
| Pool 4 | 10 | 0.372 | C1+C2+C3+C4+C5 | | | | 46.5 | | | 465 | |
| Pool 5 | 10 | 0.351 | C6+C7+C8+C9+C10 | | | | 43.875 | | | 438.75 | |
| Pool 6 | 12 | 0.057 | C11+C12+D1+D2+D3+D4 | | | | 7.125 | | | 85.5 | |
| Pool 7 | 18 | 0.024 | D5+D6+D7+D8+D9+D10+D11+D12+E1 | | | | 3 | | | 54 | |
|  | 18 | 0 | E2+E3+E4+E5+E6+E7+E8+E9+E10 | | | | 0 | | | 0 | |
| Pool 8 | 28 | 0 | E11+E12+F1+F2+F3+F4+F5+F6+F7+F8+F9+F10+F11+ F12 | | | | 0 | | | 0 | |
|  |  |  | **Total Recovered Polysaccharide** | | | | | | | **1808.25** | |
| Polysaccharide Group | Polysaccharide Content/Vial, µg | Polysaccharide Content in 40 Vials, µg | Polysaccharide Content Loaded, µg | | | Recovered polysaccharide in elution before Kd 0.5, µg | *Recovered polysaccharide in elution before Kd 0.5, % | | | Complies BP Requirement | |
| Y | 65.154 | 2606.16 | 2084.93 | | | 1808.25 | 86.73 | | | Yes | |

| **Batch: 15004**  **Group A:** | | | | | | | | | | | | | | | | | | | | | | | | | | |
| --- | --- | --- | --- | --- | --- | --- | --- | --- | --- | --- | --- | --- | --- | --- | --- | --- | --- | --- | --- | --- | --- | --- | --- | --- | --- | --- |
| **Pool Number** | **Pool Volume, mL** | | | | | | | **Polysaccharide, µg/8µl** | | | **Fractions Included** | | | | | | **Polysaccharide, µg/mL** | | | | | | | | | **Total Polysaccharide, µg** |
| Pool 1 | 28 | | | | | | | 0 | | | A1+A2+A3+A4+A5+A6+A7+A8+A9+A10+A11+A12+B1+B2 | | | | | | 0 | | | | | | | | | 0 |
| Pool 2 | 10 | | | | | | | 0.334 | | | B3+B4+B5+B6+B7 | | | | | | 41.75 | | | | | | | | | 417.5 |
| Pool 3 | 8 | | | | | | | 0.282 | | | B8+B9+B10+B11 | | | | | | 35.25 | | | | | | | | | 282 |
| Pool 4 | 12 | | | | | | | 0.259 | | | B12+C1+C2+C3+C4+C5 | | | | | | 32.375 | | | | | | | | | 388.5 |
| Pool 5 | 10 | | | | | | | 0.239 | | | C6+C7+C8+C9+C10 | | | | | | 29.875 | | | | | | | | | 298.75 |
| Pool 6 | 12 | | | | | | | 0.115 | | | C11+C12+D1+D2+D3+D4 | | | | | | 14.375 | | | | | | | | | 172.5 |
| Pool 7 | 18 | | | | | | | 0.06 | | | D5+D6+D7+D8+D9+D10+D11+D12+E1 | | | | | | 7.5 | | | | | | | | | 135 |
| Pool 8 | 18 | | | | | | | 0 | | | E2+E3+E4+E5+E6+E7+E8+E9+E10 | | | | | | 0 | | | | | | | | | 0 |
| Pool 9 | 28 | | | | | | | 0 | | | E11+E12+F1+F2+F3+F4+F5+F6+F7+F8+F9+F10+F11+F12 | | | | | | 0 | | | | | | | | | 0 |
|  |  | | | | | | |  | | | **Total Recovered Polysaccharide** | | | | | | | | | | | | | | | **1694.25** |
| Polysaccharide Group | Polysaccharide Content/Vial, µg | | | | | | | Polysaccharide Content in 40 Vials, µg | | | Polysaccharide Content Loaded, µg | | | Recovered polysaccharide in elution before Kd 0.5, µg | | | | | | *Recovered polysaccharide in elution before Kd 0.5, % | | | | | | Complies BP Requirement |
| A | 65.565 | | | | | | | 2622.60 | | | 2098.08 | | | 1694.25 | | | | | | 80.75 | | | | | | Yes |
| **Group C:** | | | | | | | | | | | | | | | | | | | | | | | | | | |
| **Pool Number** | | **Pool Volume, mL** | **Polysaccharide, µg/8µl** | | | | | | **Fractions Included** | | | | | | | | | | | **Polysaccharide, µg/mL** | | | | | **Total Polysaccharide, µg** | |
| Pool 1 | | 28 | 0 | | | | | | A1+A2+A3+A4+A5+A6+A7+A8+A9+A10+A11+A12+B1+B2 | | | | | | | | | | | 0 | | | | | 0 | |
| Pool 2 | | 10 | 0.389 | | | | | | B3+B4+B5+B6+B7 | | | | | | | | | | | 48.625 | | | | | 486.25 | |
| Pool 3 | | 8 | 0.309 | | | | | | B8+B9+B10+B11 | | | | | | | | | | | 38.625 | | | | | 309 | |
| Pool 4 | | 12 | 0.206 | | | | | | B12+C1+C2+C3+C4+C5 | | | | | | | | | | | 25.75 | | | | | 309 | |
| Pool 5 | | 10 | 0.191 | | | | | | C6+C7+C8+C9+C10 | | | | | | | | | | | 23.875 | | | | | 238.75 | |
| Pool 6 | | 12 | 0.117 | | | | | | C11+C12+D1+D2+D3+D4 | | | | | | | | | | | 14.625 | | | | | 175.5 | |
| Pool 7 | | 18 | 0.033 | | | | | | D5+D6+D7+D8+D9+D10+D11+D12+E1 | | | | | | | | | | | 4.125 | | | | | 74.25 | |
| Pool 8 | | 18 | 0.014 | | | | | | E2+E3+E4+E5+E6+E7+E8+E9+E10 | | | | | | | | | | | 1.75 | | | | | 31.5 | |
| Pool 9 | | 28 | 0 | | | | | | E11+E12+F1+F2+F3+F4+F5+F6+F7+F8+F9+F10+F11+F12 | | | | | | | | | | | 0 | | | | | 0 | |
|  | |  |  | | | | | | **Total Recovered Polysaccharide** | | | | | | | | | | |  | | | | | **1624.25** | |
| Polysaccharide Group | | Polysaccharide Content/Vial, µg | Polysaccharide Content in 40 Vials, µg | | | | | | Polysaccharide Content Loaded, µg | | | Recovered polysaccharide in elution before Kd 0.5, µg | | | | | | | | *Recovered polysaccharide in elution before Kd 0.5, % | | | | | Complies BP Requirement | |
| C | | 60.125 | 2405.00 | | | | | | 1924.00 | | | 1624.25 | | | | | | | | 84.42 | | | | | Yes | |
| **Group W:** | | | | | | | | | | | | | | | | | | | | | | | | | | |
| **Pool Number** | | **Pool Volume, mL** | **Polysaccharide, µg/8µl** | | | | | | | **Fractions Included** | | | | | | | | | | **Polysaccharide, µg/mL** | | | | | **Total Polysaccharide, µg** | |
| Pool 1 | | 28 | 0 | | | | | | | A1+A2+A3+A4+A5+A6+A7+A8+A9+A10+A11+A12+B1+B2 | | | | | | | | | | 0 | | | | | 0 | |
| Pool 2 | | 10 | 0.422 | | | | | | | B3+B4+B5+B6+B7 | | | | | | | | | | 52.75 | | | | | 527.5 | |
| Pool 3 | | 8 | 0.33 | | | | | | | B8+B9+B10+B11 | | | | | | | | | | 41.25 | | | | | 330 | |
| Pool 4 | | 12 | 0.237 | | | | | | | B12+C1+C2+C3+C4+C5 | | | | | | | | | | 29.625 | | | | | 355.5 | |
| Pool 5 | | 10 | 0.212 | | | | | | | C6+C7+C8+C9+C10 | | | | | | | | | | 26.5 | | | | | 265 | |
| Pool 6 | | 12 | 0.09 | | | | | | | C11+C12+D1+D2+D3+D4 | | | | | | | | | | 11.25 | | | | | 135 | |
| Pool 7 | | 18 | 0.026 | | | | | | | D5+D6+D7+D8+D9+D10+D11+D12+E1 | | | | | | | | | | 3.25 | | | | | 58.5 | |
| Pool 8 | | 46 | 0 | | | | | | | E2+E3+E4+E5+E6+E7+E8+E9+E10+E11+E12+F1+F2+F3+F4+F5+F6+F7+F8+F9+F10+F11+F12 | | | | | | | | | | 0 | | | | | 0 | |
|  | |  |  | | | | | | | **Total Recovered Polysaccharide** | | | | | | | | | |  | | | | | **1671.5** | |
| Polysaccharide Group | | Polysaccharide Content/Vial, µg | Polysaccharide Content in 40 Vials, µg | | | | | | | Polysaccharide Content Loaded, µg | | | | | | Recovered polysaccharide in elution before Kd 0.5, µg | | | | *Recovered polysaccharide in elution before Kd 0.5, % | | | | | Complies BP Requirement | |
| W | | 64.862 | 2594.48 | | | | | | | 2075.58 | | | | | | 1671.5 | | | | 80.53 | | | | | Yes | |
| **Group Y:** | | | | | | | | | | | | | | | | | | | | | | | | | | |
| **Pool Number** | **Pool Volume, mL** | | **Polysaccharide, µg/8µl** | | | | | | | **Fractions Included** | | | | | | | | | | | | **Polysaccharide, µg/mL** | **Total Polysaccharide, µg** | | | |
| Pool 1 | 28 | | 0 | | | | | | | A1+A2+A3+A4+A5+A6+A7+A8+A9+A10+A11+A12+B1+B2 | | | | | | | | | | | | 0 | 0 | | | |
| Pool 2 | 10 | | 0.405 | | | | | | | B3+B4+B5+B6+B7 | | | | | | | | | | | | 50.625 | 506.25 | | | |
| Pool 3 | 8 | | 0.325 | | | | | | | B8+B9+B10+B11 | | | | | | | | | | | | 40.625 | 325 | | | |
| Pool 4 | 12 | | 0.321 | | | | | | | B12+C1+C2+C3+C4+C5 | | | | | | | | | | | | 40.125 | 481.5 | | | |
| Pool 5 | 10 | | 0.221 | | | | | | | C6+C7+C8+C9+C10 | | | | | | | | | | | | 27.625 | 276.25 | | | |
| Pool 6 | 12 | | 0.065 | | | | | | | C11+C12+D1+D2+D3+D4 | | | | | | | | | | | | 8.125 | 97.5 | | | |
| Pool 7 | 18 | | 0.025 | | | | | | | D5+D6+D7+D8+D9+D10+D11+D12+E1 | | | | | | | | | | | | 3.125 | 56.25 | | | |
| Pool 8 | 46 | | 0 | | | | | | | E2+E3+E4+E5+E6+E7+E8+E9+E10+E11+E12+F1+F2+F3+F4+F5+F6+F7+F8+F9+F10+F11+ F12 | | | | | | | | | | | | 0 | 0 | | | |
|  |  | |  | | | | | | | **Total Recovered Polysaccharide** | | | | | | | | | | | | | **1742.75** | | | |
| Polysaccharide Group | Polysaccharide Content/Vial, µg | | Polysaccharide Content in 40 Vials, µg | | | | | | | Polysaccharide Content Loaded, µg | | | | | | Recovered polysaccharide in elution before Kd 0.5, µg | | | | | | *Recovered polysaccharide in elution before Kd 0.5, % | Complies BP Requirement | | | |
| Y | 66.337 | | 2653.48 | | | | | | | 2122.78 | | | | | | 1742.75 | | | | | | 82.10 | Yes | | | |
| **Batch: 15005**  **Group A:** | | | | | | | | | | | | | | | | | | | | | | | | | | |
| **Pool Number** | **Pool Volume, mL** | | | **Polysaccharide, µg/8µl** | | | | | | **Fractions Included** | | | | | | | | | | | **Polysaccharide, µg/mL** | | | **Total Polysaccharide, µg** | | |
| Pool 1 | 26 | | | 0 | | | | | | A1+A2+A3+A4+A5+A6+A7+A8+A9+A10+A11+A12+B1 | | | | | | | | | | | 0 | | | 0 | | |
| Pool 2 | 10 | | | 0.263 | | | | | | B2+B3+B4+B5+B6 | | | | | | | | | | | 32.625 | | | 326.25 | | |
| Pool 3 | 8 | | | 0.275 | | | | | | B7+B8+B9+B10 | | | | | | | | | | | 40.5 | | | 324 | | |
| Pool 4 | 14 | | | 0.302 | | | | | | B11+B12+C1+C2+C3+C4+C5 | | | | | | | | | | | 46.625 | | | 652.75 | | |
| Pool 5 | 10 | | | 0.194 | | | | | | C6+C7+C8+C9+C10 | | | | | | | | | | | 18.5 | | | 185 | | |
| Pool 6 | 12 | | | 0.137 | | | | | | C11+C12+D1+D2+D3+D4 | | | | | | | | | | | 14.875 | | | 178.5 | | |
| Pool 7 | 20 | | | 0.06 | | | | | | D5+D6+D7+D8+D9+D10+D11+D12+E1+E2 | | | | | | | | | | | 1.875 | | | 37.5 | | |
| Pool 8 | 44 | | | 0 | | | | | | E3+E4+E5+E6+E7+E8+E9+E10 | | | | | | | | | | | 0 | | | 0 | | |
| Pool 9 |  | | |  | | | | | | E11+E12+F1+F2+F3+F4+F5+F6+F7+F8+F9+F10+F11+F12 | | | | | | | | | | | 0 | | | 0 | | |
|  |  | | |  | | | | | | **Total Recovered Polysaccharide** | | | | | | | | | | | | | | **1704** | | |
| Polysaccharide Group | Polysaccharide Content/Vial, µg | | | Polysaccharide Content in 40 Vials, µg | | | | | | Polysaccharide Content Loaded, µg | | | Recovered polysaccharide in elution before Kd 0.5, µg | | | | *Recovered polysaccharide in elution before Kd 0.5, % | | | | | | | Complies BP Requirement | | |
| A | 63.391 | | | 2535.64 | | | | | | 2028.51 | | | 1704 | | | | 84.00 | | | | | | | Yes | | |
| **Group C:** | | | | | | | | | | | | | | | | | | | | | | | | | | |
| **Pool Number** | **Pool Volume, mL** | | | **Polysaccharide, µg/8µl** | | | | | | **Fractions Included** | | | | | | | **Polysaccharide, µg/mL** | | | | | | | **Total Polysaccharide, µg** | | |
| Pool 1 | 26 | | | 0 | | | | | | A1+A2+A3+A4+A5+A6+A7+A8+A9+A10+A11+A12+B1 | | | | | | | 0 | | | | | | | 0 | | |
| Pool 2 | 10 | | | 0.252 | | | | | | B2+B3+B4+B5+B6 | | | | | | | 31.5 | | | | | | | 315 | | |
| Pool 3 | 8 | | | 0.291 | | | | | | B7+B8+B9+B10 | | | | | | | 36.375 | | | | | | | 291 | | |
| Pool 4 | 14 | | | 0.346 | | | | | | B11+B12+C1+C2+C3+C4+C5 | | | | | | | 43.25 | | | | | | | 605.5 | | |
| Pool 5 | 10 | | | 0.249 | | | | | | C6+C7+C8+C9+C10 | | | | | | | 31.125 | | | | | | | 311.25 | | |
| Pool 6 | 12 | | | 0.112 | | | | | | C11+C12+D1+D2+D3+D4 | | | | | | | 14 | | | | | | | 168 | | |
| Pool 7 | 20 | | | 0.054 | | | | | | D5+D6+D7+D8+D9+D10+D11+D12+E1+E2 | | | | | | | 6.75 | | | | | | | 135 | | |
| Pool 8 | 16 | | | 0 | | | | | | E3+E4+E5+E6+E7+E8+E9+E10 | | | | | | | 0 | | | | | | | 0 | | |
| Pool 9 | 28 | | | 0 | | | | | | E11+E12+F1+F2+F3+F4+F5+F6+F7+F8+F9+F10+F11+F12 | | | | | | | 0 | | | | | | | 0 | | |
|  |  | | |  | | | | | | **Total Recovered Polysaccharide** | | | | | | |  | | | | | | | **1825.75** | | |
| Polysaccharide Group | Polysaccharide Content/Vial, µg | | | Polysaccharide Content in 40 Vials, µg | | | | | | Polysaccharide Content Loaded, µg | | | Recovered polysaccharide in elution before Kd 0.5, µg | | | | *Recovered polysaccharide in elution before Kd 0.5, % | | | | | | | Complies BP Requirement | | |
| C | 59.64 | | | 2385.60 | | | | | | 1908.48 | | | 1825.75 | | | | 95.67 | | | | | | | Yes | | |
| **Group W:** | | | | | | | | | | | | | | | | | | | | | | | | | | |
| **Pool Number** | **Pool Volume, mL** | | | | **Polysaccharide, µg/8µl** | | | | | **Fractions Included** | | | | | | | **Polysaccharide, µg/mL** | | | | | | | **Total Polysaccharide, µg** | | |
| Pool 1 | 26 | | | | 0 | | | | | A1+A2+A3+A4+A5+A6+A7+A8+A9+A10+A11+A12+B1 | | | | | | | 0 | | | | | | | 0 | | |
| Pool 2 | 10 | | | | 0.261 | | | | | B2+B3+B4+B5+B6 | | | | | | | 32.625 | | | | | | | 326.25 | | |
| Pool 3 | 8 | | | | 0.324 | | | | | B7+B8+B9+B10 | | | | | | | 40.5 | | | | | | | 324 | | |
| Pool 4 | 14 | | | | 0.373 | | | | | B11+B12+C1+C2+C3+C4+C5 | | | | | | | 46.625 | | | | | | | 652.75 | | |
| Pool 5 | 10 | | | | 0.148 | | | | | C6+C7+C8+C9+C10 | | | | | | | 18.5 | | | | | | | 185 | | |
| Pool 6 | 12 | | | | 0.119 | | | | | C11+C12+D1+D2+D3+D4 | | | | | | | 14.875 | | | | | | | 178.5 | | |
| Pool 7 | 20 | | | | 0.015 | | | | | D5+D6+D7+D8+D9+D10+D11+D12+E1+E2 | | | | | | | 1.875 | | | | | | | 37.5 | | |
| Pool 8 | 16 | | | | 0 | | | | | E3+E4+E5+E6+E7+E8+E9+E10 | | | | | | | 0 | | | | | | | 0 | | |
| Pool 9 | 28 | | | |  | | | | | E11+E12+F1+F2+F3+F4+F5+F6+F7+F8+F9+F10+F11+F12 | | | | | | | 0 | | | | | | | 0 | | |
|  |  | | | |  | | | | | **Total Recovered Polysaccharide** | | | | | | |  | | | | | | | **1704** | | |
| Polysaccharide Group | Polysaccharide Content/Vial, µg | | | | Polysaccharide Content in 40 Vials, µg | | | | | Polysaccharide Content Loaded, µg | | | Recovered polysaccharide in elution before Kd 0.5, µg | | | | *Recovered polysaccharide in elution before Kd 0.5, % | | | | | | | Complies BP Requirement | | |
| W | 64.347 | | | | 2573.88 | | | | | 2059.10 | | | 1704 | | | | 82.75 | | | | | | | Yes | | |
| **Group Y:** | | | | | | | | | | | | | | | | | | | | | | | | | | |
| **Pool Number** | **Pool Volume, mL** | | | | **Polysaccharide, µg/8µl** | | | | | **Fractions Included** | | | | | | | **Polysaccharide, µg/mL** | | | | | | | **Total Polysaccharide, µg** | | |
| Pool 1 | 26 | | | | 0 | | | | | A1+A2+A3+A4+A5+A6+A7+A8+A9+A10+A11+A12+B1 | | | | | | | 0 | | | | | | | 0 | | |
| Pool 2 | 10 | | | | 0.374 | | | | | B2+B3+B4+B5+B6 | | | | | | | 46.75 | | | | | | | 467.5 | | |
| Pool 3 | 8 | | | | 0.404 | | | | | B7+B8+B9+B10 | | | | | | | 50.5 | | | | | | | 404 | | |
| Pool 4 | 14 | | | | 0.273 | | | | | B11+B12+C1+C2+C3+C4+C5 | | | | | | | 34.125 | | | | | | | 477.75 | | |
| Pool 5 | 10 | | | | 0.192 | | | | | C6+C7+C8+C9+C10 | | | | | | | 24 | | | | | | | 240 | | |
| Pool 6 | 12 | | | | 0.109 | | | | | C11+C12+D1+D2+D3+D4 | | | | | | | 13.625 | | | | | | | 163.5 | | |
| Pool 7 | 20 | | | | 0.002 | | | | | D5+D6+D7+D8+D9+D10+D11+D12+E1+E2 | | | | | | | 0.25 | | | | | | | 5 | | |
| Pool 8 | 16 | | | | 0 | | | | | E3+E4+E5+E6+E7+E8+E9+E10 | | | | | | | 0 | | | | | | | 0 | | |
| Pool 9 | 28 | | | |  | | | | | E11+E12+F1+F2+F3+F4+F5+F6+F7+F8+F9+F10+F11+F12 | | | | | | | 0 | | | | | | | 0 | | |
|  |  | | | |  | | | | | **Total Recovered Polysaccharide** | | | | | | | | | | | | | | **1757.75** | | |
| Polysaccharide Group | Polysaccharide Content/Vial, µg | | | | Polysaccharide Content in 40 Vials, µg | | | | | Polysaccharide Content Loaded, µg | | | Recovered polysaccharide in elution before Kd 0.5, µg | | | | *Recovered polysaccharide in elution before Kd 0.5, % | | | | | | | Complies BP Requirement | | |
| Y | 67.086 | | | | 2683.44 | | | | | 2146.75 | | | 1757.75 | | | | 81.88 | | | | | | | Yes | | |
| **Batch: 15006**  **Group A:** | | | | | | | | | | | | | | | | | | | | | | | | | | |
| **Pool Number** | **Pool Volume, mL** | | | | | **Polysaccharide, µg/8µl** | | | | **Fractions Included** | | | | | | | | **Polysaccharide, µg/mL** | | | | | | **Total Polysaccharide, µg** | | |
| Pool 1 | 26 | | | | | 0 | | | | A1+A2+A3+A4+A5+A6+A7+A8+A9+A10+A11+A12+B1 | | | | | | | | 0 | | | | | | 0 | | |
| Pool 2 | 10 | | | | | 0.269 | | | | B2+B3+B4+B5+B6 | | | | | | | | 33.625 | | | | | | 336.25 | | |
| Pool 3 | 8 | | | | | 0.35 | | | | B7+B8+B9+B10 | | | | | | | | 43.75 | | | | | | 350 | | |
| Pool 4 | 14 | | | | | 0.313 | | | | B11+B12+C1+C2+C3+C4+C5 | | | | | | | | 39.125 | | | | | | 547.75 | | |
| Pool 5 | 10 | | | | | 0.252 | | | | C6+C7+C8+C9+C10 | | | | | | | | 31.5 | | | | | | 315 | | |
| Pool 6 | 12 | | | | | 0.108 | | | | C11+C12+D1+D2+D3+D4 | | | | | | | | 13.5 | | | | | | 162 | | |
| Pool 7 | 20 | | | | | 0.023 | | | | D5+D6+D7+D8+D9+D10+D11+D12+E1+E2 | | | | | | | | 2.875 | | | | | | 57.5 | | |
| Pool 8 | 16 | | | | | 0 | | | | E3+E4+E5+E6+E7+E8+E9+E10 | | | | | | | | 0 | | | | | | 0 | | |
| Pool 9 | 28 | | | | | 0 | | | | E11+E12+F1+F2+F3+F4+F5+F6+F7+F8+F9+F10+F11+F12 | | | | | | | | 0 | | | | | | 0 | | |
|  |  | | | | |  | | | | **Total Recovered Polysaccharide** | | | | | | | | | | | | | | **1768.5** | | |
| Polysaccharide Group | Polysaccharide Content/Vial, µg | | | | | Polysaccharide Content in 40 Vials, µg | | | | Polysaccharide Content Loaded, µg | | | | | Recovered polysaccharide in elution before Kd 0.5, µg | | | | *Recovered polysaccharide in elution before Kd 0.5, % | | | | | Complies BP Requirement | | |
| A | 64.367 | | | | | 2574.68 | | | | 2059.74 | | | | | 1768.5 | | | | 85.86 | | | | | Yes | | |
| **Group C:** | | | | | | | | | | | | | | | | | | | | | | | | | | |
| **Pool Number** | **Pool Volume, mL** | | | | | **Polysaccharide, µg/8µl** | | | | **Fractions Included** | | | | | | | | | **Polysaccharide, µg/mL** | | | | | **Total Polysaccharide, µg** | | |
| Pool 1 | 26 | | | | | 0 | | | | A1+A2+A3+A4+A5+A6+A7+A8+A9+A10+A11+A12+B1 | | | | | | | | | 0 | | | | | 0 | | |
| Pool 2 | 10 | | | | | 0.302 | | | | B2+B3+B4+B5+B6 | | | | | | | | | 37.75 | | | | | 377.5 | | |
| Pool 3 | 8 | | | | | 0.313 | | | | B7+B8+B9+B10 | | | | | | | | | 39.125 | | | | | 313 | | |
| Pool 4 | 14 | | | | | 0.279 | | | | B11+B12+C1+C2+C3+C4+C5 | | | | | | | | | 34.875 | | | | | 488.25 | | |
| Pool 5 | 10 | | | | | 0.159 | | | | C6+C7+C8+C9+C10 | | | | | | | | | 19.875 | | | | | 198.75 | | |
| Pool 6 | 12 | | | | | 0.127 | | | | C11+C12+D1+D2+D3+D4 | | | | | | | | | 15.875 | | | | | 190.5 | | |
| Pool 7 | 20 | | | | | 0.038 | | | | D5+D6+D7+D8+D9+D10+D11+D12+E1+E2 | | | | | | | | | 4.75 | | | | | 95 | | |
| Pool 8 | 16 | | | | | 0 | | | | E3+E4+E5+E6+E7+E8+E9+E10 | | | | | | | | | 0 | | | | | 0 | | |
| Pool 9 | 28 | | | | | 0 | | | | E11+E12+F1+F2+F3+F4+F5+F6+F7+F8+F9+F10+F11+F12 | | | | | | | | | 0 | | | | | 0 | | |
|  |  | | | | |  | | | | **Total Recovered Polysaccharide** | | | | | | | | |  | | | | | **1663** | | |
| Polysaccharide Group | Polysaccharide Content/Vial, µg | | | | | Polysaccharide Content in 40 Vials, µg | | | | Polysaccharide Content Loaded, µg | | | | | Recovered polysaccharide in elution before Kd 0.5, µg | | | | *Recovered polysaccharide in elution before Kd 0.5, % | | | | | Complies BP Requirement | | |
| C | 60.672 | | | | | 2426.88 | | | | 1941.50 | | | | | 1663 | | | | 85.66 | | | | | Yes | | |
| **Group W:** | | | | | | | | | | | | | | | | | | | | | | | | | | |
| **Pool Number** | **Pool Volume, mL** | | | | | | **Polysaccharide, µg/8µl** | | | **Fractions Included** | | | | | | | | | **Polysaccharide, µg/mL** | | | | | **Total Polysaccharide, µg** | | |
| Pool 1 | 26 | | | | | | 0 | | | A1+A2+A3+A4+A5+A6+A7+A8+A9+A10+A11+A12+B1 | | | | | | | | | 0 | | | | | 0 | | |
| Pool 2 | 10 | | | | | | 0.347 | | | B2+B3+B4+B5+B6 | | | | | | | | | 43.375 | | | | | 433.75 | | |
| Pool 3 | 8 | | | | | | 0.369 | | | B7+B8+B9+B10 | | | | | | | | | 46.125 | | | | | 369 | | |
| Pool 4 | 14 | | | | | | 0.268 | | | B11+B12+C1+C2+C3+C4+C5 | | | | | | | | | 33.5 | | | | | 469 | | |
| Pool 5 | 10 | | | | | | 0.205 | | | C6+C7+C8+C9+C10 | | | | | | | | | 25.625 | | | | | 256.25 | | |
| Pool 6 | 12 | | | | | | 0.156 | | | C11+C12+D1+D2+D3+D4 | | | | | | | | | 19.5 | | | | | 234 | | |
| Pool 7 | 20 | | | | | | 0.021 | | | D5+D6+D7+D8+D9+D10+D11+D12+E1+E2 | | | | | | | | | 2.625 | | | | | 52.5 | | |
| Pool 8 | 16 | | | | | | 0 | | | E3+E4+E5+E6+E7+E8+E9+E10 | | | | | | | | | 0 | | | | | 0 | | |
| Pool 9 | 28 | | | | | |  | | | E11+E12+F1+F2+F3+F4+F5+F6+F7+F8+F9+F10+F11+F12 | | | | | | | | | 0 | | | | | 0 | | |
|  |  | | | | | |  | | | **Total Recovered Polysaccharide** | | | | | | | | |  | | | | | **1814.5** | | |
| Polysaccharide Group | Polysaccharide Content/Vial, µg | | | | | | Polysaccharide Content in 40 Vials, µg | | | Polysaccharide Content Loaded, µg | | | | | Recovered polysaccharide in elution before Kd 0.5, µg | | | | *Recovered polysaccharide in elution before Kd 0.5, % | | | | | Complies BP Requirement | | |
| W | 64.703 | | | | | | 2588.12 | | | 2070.50 | | | | | 1814.5 | | | | 87.64 | | | | | Yes | | |
| **Group Y:** | | | | | | | | | | | | | | | | | | | | | | | | | | |
| **Pool Number** | **Pool Volume, mL** | | | | | | **Polysaccharide, µg/8µl** | | | **Fractions Included** | | | | | | | | | **Polysaccharide, µg/mL** | | | | | **Total Polysaccharide, µg** | | |
| Pool 1 | 26 | | | | | | 0 | | | A1+A2+A3+A4+A5+A6+A7+A8+A9+A10+A11+A12+B1 | | | | | | | | | 0 | | | | | 0 | | |
| Pool 2 | 10 | | | | | | 0.343 | | | B2+B3+B4+B5+B6 | | | | | | | | | 42.875 | | | | | 428.75 | | |
| Pool 3 | 8 | | | | | | 0.392 | | | B7+B8+B9+B10 | | | | | | | | | 49 | | | | | 392 | | |
| Pool 4 | 14 | | | | | | 0.286 | | | B11+B12+C1+C2+C3+C4+C5 | | | | | | | | | 35.75 | | | | | 500.5 | | |
| Pool 5 | 10 | | | | | | 0.187 | | | C6+C7+C8+C9+C10 | | | | | | | | | 23.375 | | | | | 233.75 | | |
| Pool 6 | 12 | | | | | | 0.103 | | | C11+C12+D1+D2+D3+D4 | | | | | | | | | 12.875 | | | | | 154.5 | | |
| Pool 7 | 20 | | | | | | 0.044 | | | D5+D6+D7+D8+D9+D10+D11+D12+E1+E2 | | | | | | | | | 5.5 | | | | | 110 | | |
| Pool 8 | 16 | | | | | | 0 | | | E3+E4+E5+E6+E7+E8+E9+E10 | | | | | | | | | 0 | | | | | 0 | | |
| Pool 9 | 28 | | | | | |  | | | E11+E12+F1+F2+F3+F4+F5+F6+F7+F8+F9+F10+F11+F12 | | | | | | | | | 0 | | | | | 0 | | |
|  |  | | | | | |  | | | **Total Recovered Polysaccharide** | | | | | | | | | | | | | | **1819.5** | | |
| Polysaccharide Group | Polysaccharide Content/Vial, µg | | | | | | Polysaccharide Content in 40 Vials, µg | | | Polysaccharide Content Loaded, µg | | | | | Recovered polysaccharide in elution before Kd 0.5, µg | | | | *Recovered polysaccharide in elution before Kd 0.5, % | | | | | Complies BP Requirement | | |
| Y | 65.385 | | | | | | 2615.40 | | | 2092.32 | | | | | 1819.5 | | | | 86.96 | | | | | Yes | | |
